# How Does a Divided Population Respond to Change?

**DOI:** 10.1371/journal.pone.0128121

**Published:** 2015-07-10

**Authors:** Murad R. Qubbaj, Rachata Muneepeerakul, Rimjhim M. Aggarwal, John M. Anderies

**Affiliations:** 1 School of Sustainability, Arizona State University, Tempe, Arizona 85287, USA; 2 Mathematical, Computational, and Modeling Sciences Center, Arizona State University, Tempe, Arizona 85287, USA; 3 School of Human Evolution and Social Change, Arizona State University, Tempe, Arizona 85287, USA; University of California-Irvine, UNITED STATES

## Abstract

Most studies on the response of socioeconomic systems to a sudden shift focus on long-term equilibria or end points. Such narrow focus forgoes many valuable insights. Here we examine the transient dynamics of regime shift on a divided population, exemplified by societies divided ideologically, politically, economically, or technologically. Replicator dynamics is used to investigate the complex transient dynamics of the population response. Though simple, our modeling approach exhibits a surprisingly rich and diverse array of dynamics. Our results highlight the critical roles played by diversity in strategies and the magnitude of the shift. Importantly, it allows for a variety of strategies to arise organically as an integral part of the transient dynamics—as opposed to an independent process—of population response to a regime shift, providing a link between the population's past and future diversity patterns. Several combinations of different populations' strategy distributions and shifts were systematically investigated. Such rich dynamics highlight the challenges of anticipating the response of a divided population to a change. The findings in this paper can potentially improve our understanding of a wide range of socio-ecological and technological transitions.

## Introduction

A key question in socioeconomic studies is how a social group might respond to a sudden shift in their environment. Such rapid shifts are widely encountered in ecological, social, economic, and political systems [1–7]. Most existing research on the shift–response relationship focuses on the characteristics of the new adapted equilibrium states (or end points) of the populations. We argue that what happens in between, i.e., the transient dynamics of the response, is of significant interest to researchers and policy makers, but that such dynamics have been understudied (but see [8]).

There has been an emerging interest in examining not only whether we will attain a low carbon economic state but also how we will get to that end state. What kinds of technologies will dominate during the transition stages and for how long? Will fossil fuels continue to dominate the energy mix, and if so, for how long? These questions are important because of the emissions trajectory associated with the transient stages (not just the end state) and its consequences on the environment. For example, using a simple earth-system model for global carbon dynamics and land use change, Anderies et al. [9] showed that the long run equilibrium state (habitable earth versus global desert) depends not only on the ultimate negotiated carbon stock target but how soon that target is achieved. The transient dynamics may also determine how the burden of transition costs is distributed among different groups, thereby determining the winners and losers as well as the social inequity of the transition.

In recent years, there has been an explosion in interest in examining the social equity implications of alternative economic growth pathways [10]. The Nobel prize winning economist, Joseph Stiglitz, for example, asks in his recent New York Times best seller book [11] whether growing inequality, and the increasing gap between haves and have-nots is a price we have to inevitably pay for economic growth: are there alternative pathways that may lead to more equitable long run outcomes? All of these questions call for the examination of how transient dynamics (by which we mean dynamics that play out on relatively fast timescales of months and years such as a financial crises or political uprising) impact potential equilibria (by which we mean quasi-stable patterns that persist on timescales of decades to centuries such as market or political structures).

In a previous work [8], Muneepeerakul et al. examined the transient dynamics of a response to a regime shift. However, that work considered a population that is more or less cohesive. In this paper, we extend that work to examine the transient dynamics when a regime shift is imposed upon a divided population. Here, we present a few motivating examples to illustrate systems of interest for our discussion and what we mean by “divided population” and “regime shift”. In some economies, two distinct economic sectors (modern and traditional) may coexist in a situation often characterized as economic dualism [12]. Based on the historical experience of early industrializers (Western Europe and the US) this co-existence was hypothesized to be a transitional phenomenon in the process of economic development [13] but recent experience from today’s developing countries suggests that this dualism may, under certain conditions, deepen and become entrenched [14]. A manifestation of this dualism is the two different agricultural patterns, namely subsistence agriculture and market-oriented agriculture, in the developing world. How would such a dualistic economy respond to the effects of climate change or rapid changes in non-farming opportunities and advances in agricultural technologies brought about by globalization [15–18]?

Alternatively, consider politics. A population in a democracy may be divided by ideologies, attitudes, and beliefs [19, 20]. A recent large scale survey carried out by the Pew Research Center found, “political polarization” to be “the defining feature of early 21st century American politics, both among the public and elected officials” [21]. The survey conducted in January through March of 2014 found that Republicans and Democrats are further apart ideologically than at any point in recent history and that this increasing ideological polarization makes political compromise more difficult. The survey also found that differences between the right and the left go beyond politics and are reflected in their sharply divided views on social issues and lifestyle choices (e.g. choice of communities to live in). All this suggests that researchers and policy analysts need to explicitly account for polarization in their understanding of how US society and political system will respond to various shocks. Surely, one can find many more systems with such characteristics that motivate similar questions. Indeed, understanding how divided populations may respond to rapid shifts accompanying climate change and globalization will help us better understand how well societies may adapt, hence enabling their anticipatory governance capacity. It will also shed light on the mechanism of emergence and disappearance of subgroups within a divided population.

Our work can be seen as integrating several different streams of literature. The motivation of our paper derives from the emerging literature on adaptation to climate change and resilience of social-ecological systems, seen in the light of the recent interest in increasing inequalities and political polarization all over the world, specifically in the US. In terms of the analytics, our work comes closest to the literature on evolutionary game theory and its application to a wide variety of ecological and socio-economic contexts, specifically that of technology diffusion (see [22] for a survey). However, in almost all of this body of work, replicator dynamics is used as a selection mechanism to explain the dominance of the optimal strategy/technology at the end stage. An important critique of this application of replicator dynamics is that it fails to explain why in the real world, at any given point of time, we see a variety of strategies or technologies co-existing, even though agents know about the single optimal strategy. In order to explain variety, researchers have either relied on some other independent mechanism or assumptions, such as bounded rationality, or imperfect knowledge of available technologies, costly experimentation or errors in process of adoption, etc. In all such cases, variety creation is modeled as a process independent of existing variety [22]. In this work we explain variety creation and sustenance as part of the transient dynamics, and thus as being integral to the process of responding to the regime shift. We also show how new variety creation is linked to past patterns of diversity in the population. To the best of our knowledge, this relation between new variety creation and past patterns of diversity has not been analyzed before. In this sense, our work makes important contributions in linking the complex dynamics of response to regime shifts (e.g., due to technological change or climate change) with the literature on the dynamics of inequality generation. Moreover, our visual simulations, through videos, makes these complex dynamics easy to comprehend for the general reader.

This paper is organized as follows. Section 1 describes the model, its assumptions and the different scenarios studied. Section 2 reports a rich array of results associated with these different scenarios. In Section 3, we discuss the results and their implications as well as present related examples and applications.

## 1 Methods

### 1.1 Replicator dynamics

Considering a continuous domain of strategies, the so-called replicator equation [23–27] can be written as

$$\frac{\partial p(s,t)}{\partial t}=p\left(s,t\right)[R\left(s,t\right)-E^{t}\left[R\right]],$$ (1)

where *p*(*s*, *t*) is the frequency distribution of strategy *s* at time *t*. The expected reward (or payoff) of those individuals using strategy *s* at time *t* is represented by the reward kernel *R*(*s*, *t*). The average reward in the population state *p*(*s*, *t*) is defined as *E* ^*t*^[*R*] = ∫*p*(*s*, *t*)*R*(*s*, *t*)*ds*. Eq (1) captures the fundamental mechanism shared by natural selection and social learning and adaptation: if users of a specific strategy perform better than the average, the frequency of that strategy increases (spreads) displacing other strategies of smaller fitness. As shall be shown shortly, with an appropriate setup, this model lends itself well to the study of population response to a shift.

### 1.2 Assumptions and relevant properties of the model

We will adopt and modify the assumptions made in [8], which were designed for the study of an *undivided* population. We briefly review the assumptions made therein in this section and will describe the modifications for this study in the next section.

**Fig 1:**
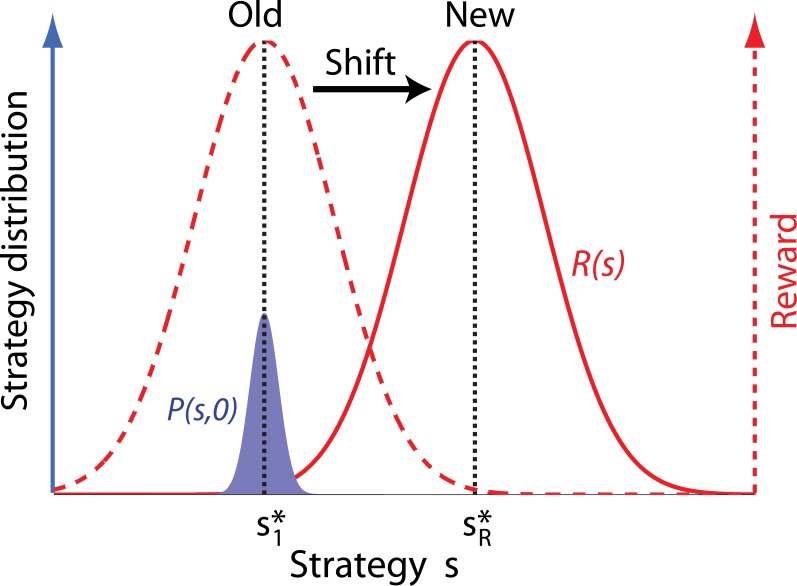
Schematic illustration of the shift-and-response scenario: The dashed and the solid red curves represent the reward kernel under the old (with a best strategy $s_{1}^{*}$) the new (with a new best strategy $s_{R}^{*}$ regimes, respectively. The blue curve represents the strategy distribution at the time that the shift occurs where it is centered about the old most popular strategy $s_{1}^{*}$.

The reward structure is assumed to be time-independent, i.e., *R*(*s*, *t*) = *R*(*s*). This is equivalent to stating that we are interested in the transient dynamics of the population right after the shift, but before the next major shift occurs. The initial strategy distribution at the time of shift, *p*_0_(*s*) = *p*(*s*, 0) is assumed to center about the best strategy of the existing regime ($s_{1}^{*}$) (in case of an undivided population; see below for the divided population) and exhibit some variation around this best strategy. While there are many possible specifications of *R*(*s*) and *p*_0_(*s*), we will assume in this study that they are of a Gaussian shape. A Gaussian *p*_0_(*s*) is seen as a reasonable and sensible approximation due to the ubiquity of the Gaussian shape in many phenomena. A Gaussian *R*(*s*), centering about the new best strategy $s_{R}^{*}$, can be written as $C\text{exp}[-{\left(s-s_{R}^{*}\right)}^{2}/2\sigma^{2}]$, where *C* > 0 is an arbitrary constant (that needs not be $1/\sqrt{2\pi }\sigma$ because *R*(*s*) is not a probability density function). Such a Gaussian *R*(*s*) corresponds to a situation in which there is a limit to how much damage a bad strategy can incur; safety net policies are an example of such a situation. Fig 1 shows a schematic illustration of this shift-and-response scenario.

Under this setting, a key finding in [8]—one that will be used as an important benchmark in the following analysis—is that if the magnitude of the shift Δs*, defined as $\left|s_{R}^{*}-s_{1}^{*}\right|$, exceeds a threshold, *p*(*s*, *t*) will divide into two groups: one corresponding to strategies around the old best strategy $s_{1}^{*}$ and the new emerging one tending to adopt strategies close to the new best strategy $s_{R}^{*}$. For the Gaussian reward kernel, this threshold, denoted by $\Delta s_{crit}^{*}$, is $3\sqrt{3}\sigma /2$. Another relevant finding is that the variance, i.e., diversity, of the initial strategy distribution determines how fast the responses is: the more diverse the strategies, the faster the response.

### 1.3 Application to a divided population

As mentioned above, the focus of the present paper is on when a regime shift is imposed upon a divided population. Accordingly, the key modification of the modeling framework described above is to employ a bimodal initial strategy distribution. Here we assume that the initial distribution, *p*_0_(*s*), can be viewed as a combination (superposition) of two distributions with peaks centering around two current “most popular” or “dominant” strategies under the current regime, $s_{1}^{*}$ and $s_{2}^{*}$, with some variation around them. We will assume that each peak is well approximated by a Gaussian distribution characterized by a variance (denoted by $D_{1}^{2}$ and $D_{2}^{2}$), presumably maintained by some fluctuation of the reward kernel under the old regime. Putting all these together, the bimodal *p*_0_(*s*) can be written as

$$p_{0}\left(s\right)=\sum_{i=1}^{2}\frac{w_{i}}{\sqrt{2\pi D_{i}^{2}}}\exp[-\frac{{\left(s-s_{i}^{*}\right)}^{2}}{2D_{i}^{2}}]$$ (2)

where *w*_*i*_ (*i* = 1, 2) represent the relative weights of the two peaks such that *w*_1_ + *w*_2_ = 1. It is critical to note here that *p*_0_(*s*) is *one bimodal distribution associated with one population, not two unimodal distributions associated with two independent populations*. In the latter case, each person can only observe and compare one’s performance to the average within one’s own group. This is *not* the situation examined in the present paper. Here, the situation is such that everyone can observe the performance of everybody else and compare one’s own performance with the average of the whole population.

### 1.4 Modeling scenarios

**Fig 2:**
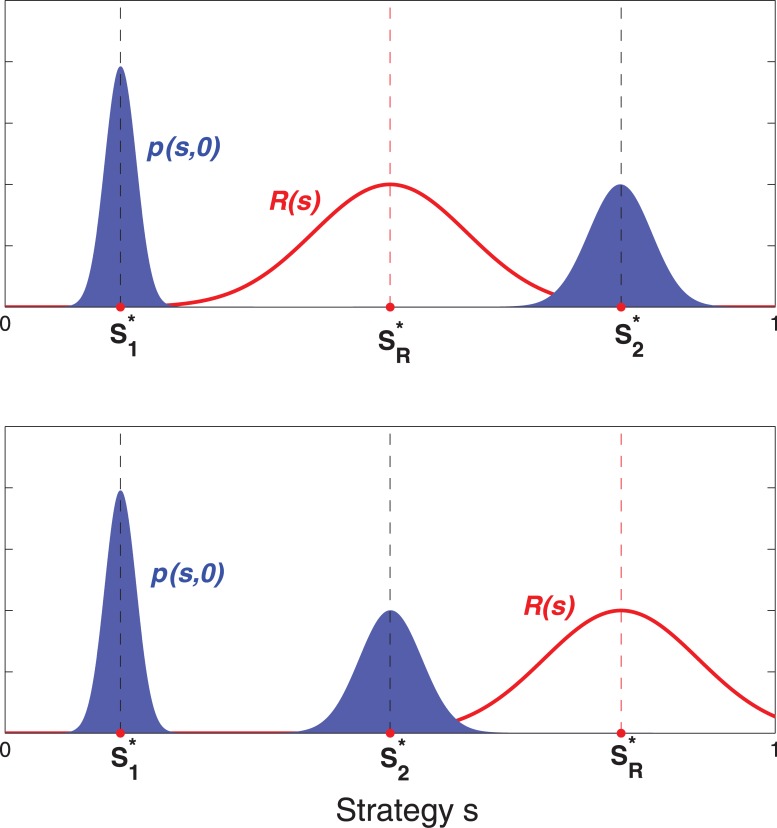
Illustration of the two different locations of the new best strategy $s_{R}^{*}$ relative to the old dominant strategies in the initial strategy distribution $s_{1}^{*}$ and $s_{2}^{*}$. a) The intermediate scenario where $s_{R}^{*}$ is located between $s_{1}^{*}$ and $s_{2}^{*}$, and b) the extreme scenario where $s_{R}^{*}$ is laterally far away from both $s_{1}^{*}$ and $s_{2}^{*}$.

Between the two peaks of *p*_0_(*s*) and the critical shift magnitude $\Delta s_{crit}^{*}$, a rich array of scenarios emerge that can be studied, depending on whether $s_{R}^{*}$ is between or outside of the range $\left[s_{1}^{*},s_{2}^{*}\right]$ and whether $\left|s_{R}^{*}-s_{1}^{*}\right|$ and/or $\left|s_{R}^{*}-s_{2}^{*}\right|$ exceeds $\Delta s_{crit}^{*}$. In addition, the symmetry (or asymmetry) between the variations around *p*_0_(*s*)’s two peaks (i.e., whether *D*_1_ and *D*_2_ are equal) exerts some influence on the dynamics of the population response and will therefore be examined. In the following analysis, we will refer to a shift with $s_{R}^{*}\in [s_{1}^{*},s_{2}^{*}]$ as a “middle-ground” shift, and a shift with $s_{R}^{*}\notin [s_{1}^{*},s_{2}^{*}]$ as an “extreme” shift. Fig 2 illustrates these two types of shifts.

## 2 Results

In the following, we will systematically investigate the effects of (i) the locations of the initial peaks with respect to the new best strategy, and (ii) the variations of these initial peaks on how a divided population responds to a sudden shift. We then show how these two aspects interplay and complicate the transient dynamics of the response. More general descriptions of typical results based on our numerical exploration under different conditions of variations *D*’s and locations of initial peaks Δs*’s are summarized in Table 1.

**Table 1:** Typical model results under different conditions of of *D*’s and Δs*’s. Recall that $s_{R}^{*}$ denotes the new best strategy. Only a subset of these results are shown and discussed in the main text; for the remaining results, the interested reader is referred to the supplementary material Text A and Videos A-Y online.

		Both $\Delta s_{1}^{*}$ and $\Delta s_{2}^{*}$ are above the threshold: $\Delta s_{1}^{*}\geq \Delta s_{2}^{*}>\Delta s_{crit}^{*}$	One Δs* is above the threshold while the other one is below: $\Delta s_{1}^{*}>\Delta s_{crit}^{*}>\Delta s_{2}^{*}$	Both $\Delta s_{1}^{*}$ and $\Delta s_{2}^{*}$ are below the threshold: $\Delta s_{crit}^{*}>\Delta s_{1}^{*}\geq \Delta s_{2}^{*}$
Middle-ground shift	***D*_1_ = *D*_2_**	Two new peaks may emerge, i.e., four peaks may coexist temporarily. When one of the old peaks is too far away from the new best strategy $s_{R}^{*}$ compared to the other, only one new peak emerges, and three peaks coexist.	The peak below the threshold moves cohesively and always dominates due to its greater reward. A new peak may not emerge if $s_{1}^{*}$ is too far away.	The two peaks move cohesively towards $s_{R}^{*}$. The closer peak approaches $s_{R}^{*}$ earlier and dominates due to its greater reward.
	***D*_1_ ≠ *D*_2_**	Two new peaks may emerge at different times, and four peaks coexist. If the variation around one peak is much greater than the other, only one new peak emerges, and three peaks coexist. The variations around the original peaks and the distances from $s_{R}^{*}$ determine which peak dominates.	If the peak above the threshold is of larger variation, a new peak may emerge if it is not too far away, and three peaks may coexist. No new peak emerges if it is too far from $s_{R}^{*}$. If the peak below the threshold is of larger variation, it moves cohesively and dominates; no new peak emerges.	Both peaks would move cohesively towards $s_{R}^{*}$. The variations around the original peaks and distances from $s_{R}^{*}$ determine which peak dominates.
Extreme shift	***D*_1_ = *D*_2_**	A new peak emerges between $s_{R}^{*}$ and the closer old most popular strategy and dominates. Three peaks may coexist.	The closer peak moves cohesively towards $s_{R}^{*}$ and keeps growing and eventually dominates. The farther peak collapses. No new peak emerges.	The closer peak moves cohesively towards $s_{R}^{*}$ and keeps growing and eventually dominates. The farther peak collapses. No new peak emerges.
	***D*_1_ ≠ *D*_2_**	If the farther original peak is of higher variation, one or two new peaks may emerge. The new peak closer to $s_{R}^{*}$ dominates, while the original peaks collapses. Three/four peaks may coexist at the same time. If the closer original peak is of larger variation, only one new peak emerges, and three peaks coexist.	If the farther original peak is of higher variation, the peak below the threshold moves cohesively towards $s_{R}^{*}$. A new peak may or may not emerge depending on how far the peak above the threshold (which eventually collapses) is from $s_{R}^{*}$. This new peak may emerge even after the complete collapse of the old peak above the threshold. If the variation around the closer original peak is sufficiently large, no new peak emerges.	If the farther original peak is of higher variation, the peak below the threshold moves cohesively towards $s_{R}^{*}$. The farther peak collapses. No new peak emerges. If the difference between the two variations is relatively large, however, a third peak may emerge close to $s_{R}^{*}$ after the collapse of the farther one and eventually dominates. If the variation around the closer original peak is sufficiently large, no new peak emerges.

Note that since the primary focus of this paper is on the transient dynamics, the reader is strongly encouraged to consider the video clips available in the supplementary material online (S1 File) in conjunction with the analysis here (some snapshots from the video clips are shown here). Note also that the critical threshold $\Delta s_{crit}^{*}$ is based on the response of an undivided (single-peak) population, as derived in [8]. It is included in the figures for comparison purposes.

### 2.1 Effects of locations of initial peaks relative to the new best strategy

In this section, we assume that variations around the two peaks are equal, i.e., *D*_1_ = *D*_2_, so that the effects of the initial peak locations are clear. Recall from Section 1.2 that *D* ^2^ controls the pace of response, and thus the two initial peaks are in this sense equally responsive in this setting. It is therefore the distances from the new strategy, namely $\Delta s_{1}^{*}$ and $\Delta s_{2}^{*}$, that determine the response dynamics.

#### 2.1.1 Middle-ground shift

**Fig 3:**
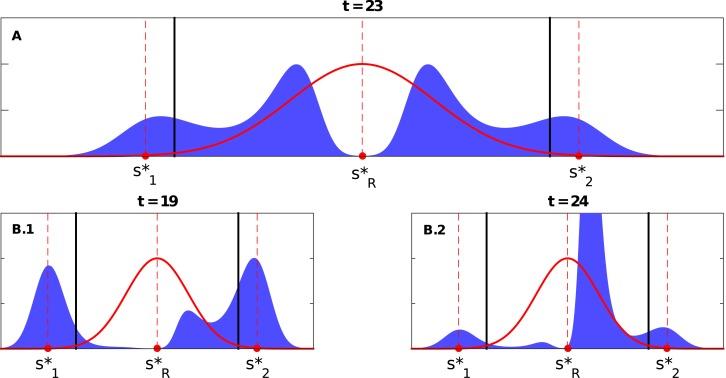
A) The panel illustrates the coexistence of four peaks at some time (*t* = 23) during the population response to a sudden middle-ground shift when $\Delta s_{1}^{*}=\Delta s_{2}^{*}=0.3>\Delta s_{crit}^{*}=0.2598$ (where $s_{1}^{*}=0.2$, $s_{2}^{*}=0.8$, $s_{R}^{*}=0.5$, and *σ* = 0.1 with *D*_1_ = *D*_2_ = 0.04), see Video A in S1 File. B) The bottom panels illustrates the different number of coexisting peaks at different times (*t* = 19 and 24) for the case when $\Delta s_{1}^{*}=0.35>\Delta s_{2}^{*}=0.32>\Delta s_{crit}^{*}=0.2598$ (where $s_{1}^{*}=0.15$, $s_{2}^{*}=0.82$, $s_{R}^{*}=0.5$, and *σ* = 0.1 with *D*_1_ = *D*_2_ = 0.045), (see Video B in S1 File). Note that both cases are when the variations of the two peaks of the initial population distribution are equal to each other, i.e., *D*_1_ = *D*_2_. The dashed lines show the locations of $s_{1}^{*}$, $s_{2}^{*}$, and $s_{R}^{*}$, while the solid lines represent the the theoretically calculated threshold(s) for the single peak population distribution case, i.e., $\Delta s_{crit}^{*}=3\sqrt{3}\sigma /2$.

When both old dominant strategies are of equal distances from the new best one and above the critical threshold, i.e., $\Delta s_{1}^{*}=\Delta s_{2}^{*}>\Delta s_{crit}^{*}$, we observe that two new peaks appear simultaneously; as a result, four peaks coexist for a short period of time before the old peaks disappear and the two new emerging peaks approach the new best strategy to form one single peak in the limit (see Fig 3A and Video A in S1 File). In this case, the initial peaks seem to behave *as if* they were two independent populations.

Breaking the symmetry in Δs* but keeping them above the threshold, say, $\Delta s_{1}^{*}>\Delta s_{2}^{*}>\Delta s_{crit}^{*}$—the $s_{2}^{*}$ peak is closer to $s_{R}^{*}$ and thus of higher payoff than that of $s_{1}^{*}$—we still observe the emergence of two new peaks, but not simultaneously. As a result, four or three peaks may be observed during the transition period. The peak emerging between $s_{R}^{*}$ and $s_{2}^{*}$ is formed first and quickly becomes the dominant peak. Both new peaks continue to approach towards the new best strategy before they unite at the neighborhood of $s_{R}^{*}$ (see Fig 3B.1 and 3B.2; and Video B in S1 File). If $\Delta s_{1}^{*}$ is too large, the second new peak does not emerge at all, and only three peaks, at most, coexist at a given time (see Video C in S1 File).

What happens if $\Delta s_{1}^{*}>\Delta s_{crit}^{*}>\Delta s_{2}^{*}$, i.e., only one peak is located beyond the critical threshold? The $s_{2}^{*}$ peak would move cohesively towards $s_{R}^{*}$, while a new peak between $s_{R}^{*}$ and $s_{1}^{*}$ may or may not emerge, depending on how large $\Delta s_{1}^{*}$ is. If $\Delta s_{1}^{*}$ is too large, the $s_{1}^{*}$ peak would simply disappear (see Video D in S1 File); if $s_{1}^{*}$ is not too far from $s_{R}^{*}$, a new peak will emerge, but it may not grow significantly as it approaches $s_{R}^{*}$ due to the dominance of the peak cohesively moving from $s_{2}^{*}$ (see Video E in S1 File). If both peaks are located within the critical threshold, i.e., $\Delta s_{1}^{*},\Delta s_{2}^{*}<\Delta s_{crit}^{*}$, both will move cohesively towards $s_{R}^{*}$. If $\Delta s_{1}^{*}=\Delta s_{2}^{*}$, it will take them a very long time for the two peaks to merge completely (see Video F in S1 File). However, if one is closer than the other, it will reach $s_{R}^{*}$’s neighborhood earlier and dominate at the end (see Video G in S1 File).

#### 2.1.2 Extreme shift

**Fig 4:**
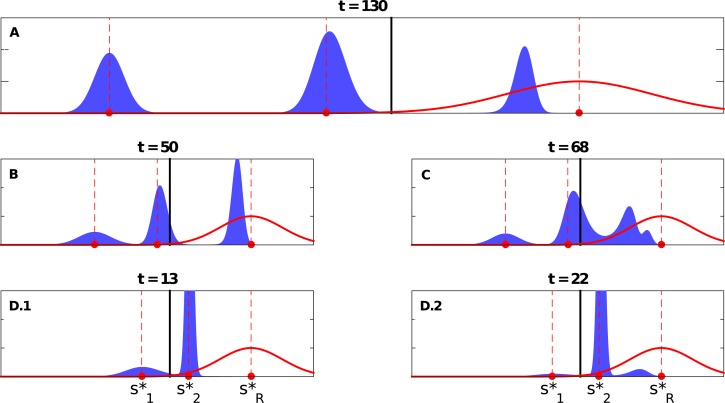
Snapshots of the coexisting peaks for the extreme case. A) The top panel shows the coexistence of three peaks when $\Delta s_{1}^{*}=0.65,\Delta s_{2}^{*}=0.35>\Delta s_{crit}^{*}=0.2598,$ and with *symmetric* variations, i.e., *D*_1_ = *D*_2_ = 0.2 (where $s_{1}^{*}=0.15$, $s_{2}^{*}=0.45$, $s_{R}^{*}=0.8$, *σ* = 0.1), see Video O in S1 File. B) and C) Three or four peaks may coexist when $\Delta s_{1}^{*}=0.5,\Delta s_{2}^{*}=0.3>\Delta s_{crit}^{*}=0.2598$ but with *asymmetric* variations, i.e., *D*_1_ > *D*_2_ (where $s_{1}^{*}=0.3$, $s_{2}^{*}=0.5$, $s_{R}^{*}=0.8$, *σ* = 0.1, with *D*_1_ = 0.05 and *D*_2_ = 0.02 for (B), and *D*_1_ = 0.0420 and *D*_2_ = 0.0225 for (C)), see R and S Videos in S1 File, respectively. The bottom panels (D.1 and D.2) show the snapshots at different times when $\Delta s_{1}^{*}=0.35>\Delta s_{crit}^{*}=0.2598>\Delta s_{2}^{*}=0.2$, and with *asymmetric* variations (where $s_{1}^{*}=0.45$, $s_{2}^{*}=0.6$, $s_{R}^{*}=0.8$, *σ* = 0.1, with *D*_1_ = 0.05 and *D*_2_ = 0.01), see Video U in S1 File. The dashed lines show the locations of $s_{1}^{*}$, $s_{2}^{*}$, and $s_{R}^{*}$, while the solid lines represent the the theoretically calculated threshold(s) for the single peak population distribution case, i.e., $\Delta s_{crit}^{*}=3\sqrt{3}\sigma /2$.

We now consider the case of extreme shift, i.e., $s_{R}^{*}\notin [s_{1}^{*},s_{2}^{*}]$; without loss of generality, we assume that $s_{1}^{*}<s_{2}^{*}<s_{R}^{*}$ (see Fig 2B). Consequently, $\Delta s_{2}^{*}<\Delta s_{1}^{*}$ in what follows. When both peaks are above the threshold ($\Delta s_{1}^{*},\Delta s_{2}^{*}>\Delta s_{crit}^{*}$), a new peak emerges between $s_{2}^{*}$ and $s_{R}^{*}$, grows quickly, and moves toward $s_{R}^{*}$, while the original peaks disintegrate. The fast growth of this new peak seems to prevent the emergence of any additional peaks. Hence, at most three peaks can coexist at the same time (see Fig 4A; and Video O in S1 File). When $\Delta s_{2}^{*}<\Delta s_{crit}^{*}<\Delta s_{1}^{*}$, the closer peak (i.e., the $s_{2}^{*}$ peak) moves cohesively towards $s_{R}^{*}$ and keeps growing and eventually dominates (see Video P in S1 File). Similar dynamics can also be observed when $\Delta s_{1}^{*},\Delta s_{2}^{*}<\Delta s_{crit}^{*}$ (see Video Q in S1 File).

### 2.2 Effects of variation (diversity) around the initial peaks

We now consider the case where $s_{1}^{*}$ and $s_{2}^{*}$ are of equal distance from the new best strategy $s_{R}^{*}$, i.e., $\Delta s_{1}^{*}=\Delta s_{2}^{*}$, and investigate the effects of the variation *D* around the initial peaks. Note that this condition $\Delta s_{1}^{*}=\Delta s_{2}^{*}$ is by definition only possible in cases with a middle-ground shift. Without loss of generality and for ease of discussion, in the followings, let us suppose that *D*_1_ > *D*_2_; we can accordingly expect that the $s_{1}^{*}$ peak would respond faster than its $s_{2}^{*}$ counterpart.

**Fig 5:**
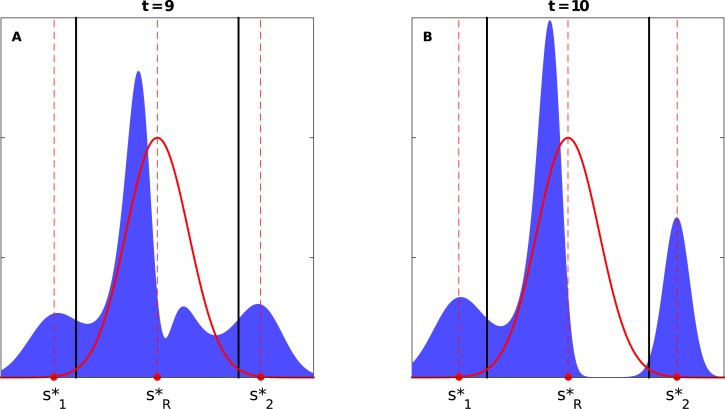
Snapshots show the coexistence of different peaks in the case of asymmetric variations of the peaks in the initial population distribution (*D*_1_ > *D*_2_) and when $\Delta s_{1}^{*}=\Delta s_{2}^{*}>\Delta s_{crit}^{*}$. A) Four peaks may coexist when *D*_1_ > *D*_2_ ($\Delta s_{1}^{*}=\Delta s_{2}^{*}=0.33$ and $\Delta s_{crit}^{*}=0.2589$, where $s_{1}^{*}=0.17$, $s_{2}^{*}=0.83$, $s_{R}^{*}=0.5$, and *σ* = 0.1, with *D*_1_ = 0.08 and *D*_2_ = 0.07, see Video H in S1 File). B) When the asymmetry in variations is stronger, i.e., *D*_1_ > > *D*_2_, only three peaks coexist ($\Delta s_{1}^{*}=\Delta s_{2}^{*}=0.35$ and $\Delta s_{crit}^{*}=0.2589$, where $s_{1}^{*}=0.15$, $s_{2}^{*}=0.85$, $s_{R}^{*}=0.5$, and *σ* = 0.1, with *D*_1_ = 0.08 and *D*_2_ = 0.04, see Video I in S1 File). The dashed lines show the locations of $s_{1}^{*}$, $s_{2}^{*}$, and $s_{R}^{*}$, while the solid lines represent the the theoretically calculated threshold(s) for the single peak population distribution case, i.e., $\Delta s_{crit}^{*}=3\sqrt{3}\sigma /2$.

When $\Delta s_{1}^{*}=\Delta s_{2}^{*}>\Delta s_{crit}^{*}$, four peaks may be observed during the transition period. The new emerging peaks appear at different times; the first would emerge between $s_{R}^{*}$ and $s_{1}^{*}$ and dominate over the peak that appears later between $s_{R}^{*}$ and $s_{2}^{*}$ (see Fig 5A; and Video H in S1 File). However, if *D*_1_ ≫ *D*_2_, the first peak would grow quickly and move towards $s_{R}^{*}$. At this point, this new peak is of both better payoff and greater frequency; this suppresses the emergence of a second new peak as agents would more likely adopt the strategies close to this new peak. As a result, only three peaks, at most, would coexist during the transition period (see Fig 5B; and Video I in S1 File). When $\Delta s_{1}^{*}=\Delta s_{2}^{*}<\Delta s_{crit}^{*}$, both peaks would move cohesively towards the new best strategy. The peak of larger value of *D* dominates.

### 2.3 Asymmetric variations and initial locations

In a general case in which there are no restrictions on the locations of the initial peaks and the variations around them, there are numerous possible dynamical scenarios. Here we discuss only a few examples to demonstrate how the dynamics becomes rich and difficult to predict or even counter-intuitive at times. Many more examples can be found in the supplementary material with videos and figures.

#### 2.3.1 Middle-ground shift

**Fig 6:**
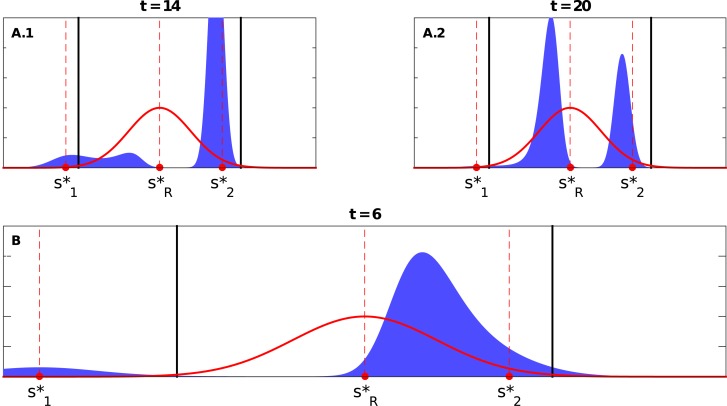
Snapshots show different number of coexisting peaks when $\Delta s_{1}^{*}>\Delta s_{crit}^{*}>\Delta s_{2}^{*}$ and *D*_1_ > *D*_2_. Top panels (A.1) show the coexistence of three peaks at *t* = 14 and (A.2) shows how the newly emerging peak near $s_{R}^{*}$ dominates over the moving peak originated near $s_{2}^{*}$ at *t* = 20, (where $\Delta s_{1}^{*}=0.3$, $\Delta s_{2}^{*}=0.2$, $\Delta s_{crit}^{*}=0.2589$, with $s_{1}^{*}=0.2$, $s_{2}^{*}=0.7$, $s_{R}^{*}=0.5$, and *σ* = 0.1, with *D*_1_ = 0.05 and *D*_2_ = 0.02, see Video J in S1 File. B) When $\Delta s_{1}^{*}$ is too large, the the moving peak originated near $s_{2}^{*}$ will dominate and no emergence of a new peak near $s_{R}^{*}$ (where $\Delta s_{1}^{*}=0.45$, $\Delta s_{2}^{*}=0.2$, $\Delta s_{crit}^{*}=0.2589$, with $s_{1}^{*}=0.05$, $s_{2}^{*}=0.7$, $s_{R}^{*}=0.5$, and *σ* = 0.1, with *D*_1_ = 0.08 and *D*_2_ = 0.06. see Video K in S1 File). The dashed lines show the locations of $s_{1}^{*}$, $s_{2}^{*}$, and $s_{R}^{*}$, while the solid lines represent the the theoretically calculated threshold(s) for the single peak population distribution case, i.e., $\Delta s_{crit}^{*}=3\sqrt{3}\sigma /2$.

Consider the case in which $\Delta s_{1}^{*}>\Delta s_{crit}^{*}>\Delta s_{2}^{*}$ and *D*_1_ > *D*_2_. In this case, the $s_{2}^{*}$ peak starts to move cohesively and more slowly (due to a lower *D*) towards $s_{R}^{*}$. This moving peak *temporarily* dominates the strategy space while a new peak is emerging between $s_{R}^{*}$ and $s_{1}^{*}$. However, the new peak may emerge very close to $s_{R}^{*}$—corresponding to better payoff—and eventually take over the moving $s_{2}^{*}$ peak (Fig 6). The maximum of three peaks is observed in this case (see Fig 6A.1 and 6A.2; and Video J in S1 File). Now, If $\Delta s_{1}^{*}$ is too large, there would be sufficient time for the moving peak from $s_{2}^{*}$ to reach the high-payoff neighborhood of $s_{R}^{*}$ such that the emergence of a new peak is suppressed (see Fig 6B; and Video K in S1 File). In other words, there are no emerging peaks at all in this case.

#### 2.3.2 Extreme shift

Consider the case when $\Delta s_{2}^{*}<\Delta s_{crit}^{*}<\Delta s_{1}^{*}$ and *D*_1_ > *D*_2_. When $\Delta s_{1}^{*}$ is relatively large, the $s_{1}^{*}$ peak quickly erodes, while the $s_{2}^{*}$ moves cohesively towards $s_{R}^{*}$ and keeps growing in the process. In this case, no new peak is observed (see Video T in S1 File). But if $\Delta s_{1}^{*}$ is just above $\Delta s_{crit}^{*}$, we may observe a new peak emerging between $s_{2}^{*}$ and $s_{R}^{*}$, while the $s_{2}^{*}$ peak moves slowly toward $s_{R}^{*}$. For a brief period of time, three peaks coexist, before the new emerging peak becomes dominant and the original peaks collapse (see Fig 4D.1 and 4D.2; and Video U in S1 File).

**Fig 7:**
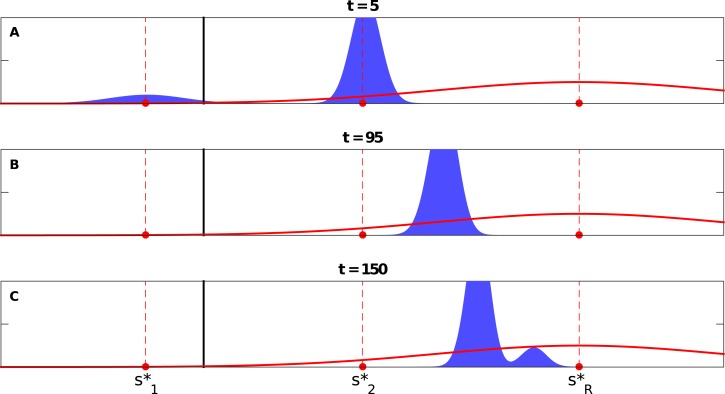
Different snapshots at different times for the case of $\Delta s_{1}^{*}>\Delta s_{crit}^{*}>\Delta s_{2}^{*}$ and with asymmetric variations (where $\Delta s_{1}^{*}=0.6$, $\Delta s_{2}^{*}=0.3$, $\Delta s_{crit}^{*}=0.5196$, with $s_{1}^{*}=0.2$, $s_{2}^{*}=0.5$, $s_{R}^{*}=0.8$, *σ* = 0.2, with *D*_1_ = 0.05 and *D*_2_ = 0.02, see Video V in S1 File). Note that the peak initially around $s_{1}^{*}$ disintegrates completely while the other peak dominates temporarily before a new peak emerges suddenly and dominates at the end. The dashed lines show the locations of $s_{1}^{*}$, $s_{2}^{*}$, and $s_{R}^{*}$, while the solid lines represent the the theoretically calculated threshold(s) for the single peak population distribution case, i.e., $\Delta s_{crit}^{*}=3\sqrt{3}\sigma /2$.

Alternatively, it is also possible that the $s_{1}^{*}$ peak collapses completely, leaving the $s_{2}^{*}$ peak to be the only peak—*temporarily*—growing and drifting toward $s_{R}^{*}$. A new peak, however, suddenly appears even closer to $s_{R}^{*}$ than the drifting one (see Fig 7 and Video V in S1 File). This emerging peak grows rapidly and becomes the dominant one, and the moving $s_{2}^{*}$ peak eventually collapses. Accordingly, in this case, we observe at most two peaks at a given time—with a brief period with only one dominant group of strategies.

## 3 Discussion

In this paper we show that a rich array of different transient dynamics are possible when a divided population is exposed to a shock. The large and growing empirical literature on adaptation to climate change from around the world bears testimony to this wide diversity in response patterns [28]. This literature has pointed to a large suite of contextual, policy, and human behavioral characteristics as possible determinants of this diversity, but has not systematically investigated how this diversity arises or what it implies for system change. Our simple model is a modest attempt to focus on just a few features of a very complex process of population response. However, it is striking that even our very simple model—with a rudimentary characterization of the selection mechanism in the form of replicator dynamics, and the assumption of complete access to information and uniform capacity to respond—yields a surprisingly rich set of transient patterns, which have largely been ignored in the formal modeling literature with its exclusive focus on end states. It is important to note that this richness is not imposed onto the model, but arises–unexpectedly, in fact–from our few simple assumptions. This is both strength of and a lesson from this model: a wide array of complex dynamics can originate from a few simple mechanisms.

Indeed, such richness makes it difficult to anticipate the dynamical consequences of a rapid change, be that natural or man-made. Our results also emphasize the important roles played by the variations around the old dominant strategies (*D*) and how far these old strategies are from the new optimal strategy Δs* (see Table 1 for a summary of the general trends and behaviors of the model). Note that only some results are shown in the main text; more results are available in the supplementary material Text A. It is worth mentioning here that our obtained results are based on numerical calculations. These results emphasize the rich dynamics of how a divided population might respond to a rapid change. This richness stresses the importance of analytical treatment of this problem (e.g., analytical expressions of the conditions for having 2 or 3 or 4 peaks during the transition). However, such analytical treatment is far from straightforward and beyond the scope of this manuscript, and is left for future work. An important result in our previous paper [8], which was based on the assumption of an initially undivided population, was that if the magnitude of the shift in dominant strategy exceeds a certain threshold (for a Gaussian reward kernel, this threshold $\Delta s_{crit}^{*}$ is $3\sqrt{3}\sigma /2$), the population will divide into two groups: one corresponding to strategies around the old most popular strategy and the new (emerging) one tending to adopt strategies close to the new best strategy. In the present work, where we consider an initially polarized population, we find that only in the special case where the variance of two subgroup populations are equal and the new best strategy lies between the two earlier most popular strategies and above the critical threshold level, do the two subgroups behave as if they were two independent populations and split in the manner reported in [8]. In almost all other cases, the response is diverse and much less predictable. In many cases, the number of new emerging peaks is less than what would be expected when the subgroups are independent. In a sense, the presence of two subgroups possibly offers greater information to others in the whole population, thus avoiding the “trial and error” of an additional peak. This suggests that in a divided population setting, with multiple existing dominant strategies, subsequent regime shifts induce fewer new peaks or lower duration of new peaks. This is an interesting result related to the role that current diversity plays in agents’ future learning in the process of adaptation.

Unfortunately there is hardly any empirical literature that is based on real time data on how different strategies or technologies diffuse in response to specific shocks, and so thus far we have not been able to formally test the predictions of our model. This is a fertile area for future research, specifically given the growing availability of large data, including the compilation of income and wealth datasets [29]. For now, it would suffice to discuss some stylized facts that have emerged from the scattered empirical literature and relate it to our model predictions. In the climate adaptation literature, diversity of beliefs, values and approaches has been seen to play a positive role in the adaptation process (see for instance, [30] for a survey). Our modeling helps further elucidate this role. Specifically, the model suggests that we cannot assume, in general, that diversity will play a positive role as society adapts to change. Rather, its role depends both on the nature of the change and the distributions of beliefs in society. In some cases, diversity may bring society together to effectively cope with change. In other (relatively rare) cases, it may lead to increased fragmentation of beliefs, at least initially. In a political system, this fragmentation of strategies (political views on the correct action to take vis-à-vis a climate change shock, for example) may generate ideological divides, generate social tension and make compromise difficult. This situation, in turn, may prolong decision-making processes and cost society valuable time (c.f. [9]), thus reducing adaptive capacity. In ecological systems, such division in strategies may be linked to biological diversity and may thus be preferred. Depending on the context, the emergence of multiple peaks (i.e., diversity) may or may not be desirable. Our model—by showing how diversity emerges, what forms it takes (number and types of peaks), and for how long—can be of potential value to a wide range of contexts exemplified above.

A potential application of our work may be to improve our understanding of different socio-ecological and technological transitions. Consider, more specifically, energy transitions. With the looming threat of climate change and the increasing recognition of the unsustainability of fossil fuel based economic systems, there is interest in examining how a transition to a renewable energy-based system can be enabled. In earlier literature, the concept of energy ladder was often used to explain how historically households have switched from biomass-based fuels, such as wood and dung, which are cheaper but also more deleterious to human heath and the environment, to mineral and hydro-power based electricity, as per capita incomes have increased [31, 32]. An important research challenge in this field has been to explain why the phenomena of “fuel stacking”, i.e. co-existence of multiple energy sources in household and regional energy portfolios, has been observed more often than “fuel switching and replacement,” as generally suggested by existing models of technology innovation and competition [33, 34]. As Stirling [35] observes, the concept of technological diversity and the coexistence of technologies is probably the least explored aspect of technological dynamics.

Our work, by formally analyzing the transition process, explicitly lays out how diversity may organically arise as part of the technology diffusion dynamics, especially in response to certain shocks. A case in point is the oil shock in the 70’s, which invoked different responses in different countries: some invested more in the oil industry; others sought a diverse portfolio with renewable alternatives. Countries in the former category may be viewed as being unimodal in their energy strategy distribution, while countries in the latter category may be viewed as having a divided set of energy strategies. Fast forward to the present day wherein more innovative energy strategies are needed to cope with climate change, with the new best strategies probably being quite different from existing ones—a situation akin to an extreme-shift scenario—our results suggest that numerous different responses are possible, which would depend on existing diversity of strategies and the payoff distribution associated with the energy mix. Our model points to some underlying mechanisms and provides potential guidance that may enable us to anticipate these responses better. For example, one may attempt to manipulate the shape of the reward kernel (e.g., through tax incentives, penalties, public relation campaigns, etc.) to exploit asymmetry and induce convergence of views/strategies rather than social tension. Additionally, other dynamics that may occur at different time scales may be affected by these different transient dynamics.

Although our characterization of the diffusion process is simple, it helps focus on the dynamics of a reasonable selection mechanism (i.e, replicator dynamics) by which specific technologies/strategies come to be adopted and /or replaced. It remains an empirical question as to what kinds of technological processes, under what kinds of socio-economic contexts, can be represented by such a selection mechanism. We hope that future studies will investigate this question and generate detailed data on the transition processes within different populations to test for the predictions of this model. All in all, as shown in section 2 and the supplementary results, the interplay between the locations of and the variations around the initial peaks yields a rich suite of possible dynamics during the transition period. These results highlight the challenges in anticipatory governance/management of a divided population in that it is simply difficult to anticipate the consequences of implementing a change. We, nonetheless, anticipate that the findings in the paper will contribute to advancing the theoretical foundation of sound anticipatory governance of social and ecological systems.

## Supporting Information

S1 File Supplementary material that includes **Text A** in which more examples of the obtained results are presented with reference to the corresponding video clips (**A-Y Video**) with their captions. (ZIP)
