# Supplementary material for: How Does a Divided Population Respond to Change?
Source: PLoS One. 2015 Jul 10;10(7):e0128121. doi: 10.1371/journal.pone.0128121 (PMC4498743; doi:10.1371/journal.pone.0128121)
Supplement: S1 File — (ZIP) [file pone.0128121.s001.zip › S1 File/Text A.pdf]

Supplementary Information:

How Does a Divided Population Respond to Change?

Murad R. Qubbaj,[1] Rachata Muneepeerakul,[1,2] Rimjhim M. Aggarwal,[1] John M. Anderies,[1,3,4]

[1]School of Sustainability,

[2]Mathematical, Computational, and Modeling Sciences Center,

[3]School of Human Evolution and Social Change, and

[4]Center for the Study of Institutional Diversity

Arizona State University, Tempe, AZ, 85287, USA

1 Review of Muneepeerakul's Model

In general, replicator dynamics can be used to study the population response to a change or shift. For a continuous domain of strategies which implies that the population is infinitely large, evolutionary game theory proposes the so-called replicator equation [1–6] defined as

$$\frac{\partial p(s,t)}{\partial t} = p(s,t)\left[R(s,t) - E^t[R]\right], \tag{1}$$

where $p(s,t)$ is the frequency distribution of strategy s at time t. This differentiable function of time t can be viewed as the state of the population or the probability density function (pdf). The expected reward (or payoff) of those individuals using strategy s at time t is represented by the reward kernel $R(s,t)$. The average reward in the population state $p(s,t)$ is defined as $E^t[R] = \int p(s,t)R(s,t)ds$ [2, 4, 7, 8]. Eq.(1) mathematically translates the fundamental principle of natural selection: if a strategy, or the users of a specific strategy, perform better than the average, the frequency of that strategy increases (spreads)

displacing other strategies of smaller fitness [7, 8]. A solution to Eq.(1) may take the form of time-dependent Boltzmann distribution [2, 3] given as follows

$$p(s,t) = p_0(s)\exp[\Phi(s,t)]/Z(t), \quad (2)$$

where $p_0(s) = p(s,0)$, i.e., the initial frequency distribution of strategy s, $\Phi(s,t) = \int_0^t R(s,\tau)d\tau$, and $Z(t) = \int_S p_0(s)\exp[\Phi(s,t)]ds$. Muneepeerakul et al. [7] applied a graphical method using the above solution of the replicator equation to study the transient dynamics of how a population responds to a sudden shift of the regime characterized by the reward kernel. Here is a summary of their assumptions and findings:

- An initial strategy distribution $p_0(s)$ is centered about a best strategy of a long-standing regime, i.e., s_1^*. This initial distribution has a variance around this best strategy. This variance which represents the diversity of strategies around the best one might be due to some fluctuations of the reward kernel under the old regime. At some instant of time, the regime characterized by a time-independent reward kernel $R(s,t) = R(s)$, momentarily shifts such that the new regime will have a new best strategy, s_R^*. Consequently, the population starts responding to this shift to adapt to the new regime such that the population distribution $p(s,t)$ evolves towards s_R^*. See Fig.1 for a schematic illustration of the shift-and-response scenario.

- For a Gaussian-like reward kernel, i.e., that is bounded from below, if the difference between the old and the new best strategies, $\Delta s^* = |s_R^* - s_1^*|$ is below a specific threshold, the population would move towards the new strategy cohesively as a traveling peak. If the difference is above the threshold, the population distribution divides into two groups–one tending to hold on the old best strategy s_1^* and the new emerging one tending to adopt the new best one s_R^* [7]. The mathematical form of this threshold can be calculated analytically for several time-independent Gaussian-like reward kernels. The results show that the threshold is mainly proportional to the parameter denoting the width of the reward kernel. For example, for a Gaussian reward kernel, i.e., $R(s) = \exp[-(s - s_R^*)^2/2\sigma^2]$, the threshold is $\Delta s_{crit}^* = 3\sqrt{3}\sigma/2$, where σ represents the standard deviation of the reward kernel.

- For reward kernels that are not bounded from below, like that of the inverted parabola, where the

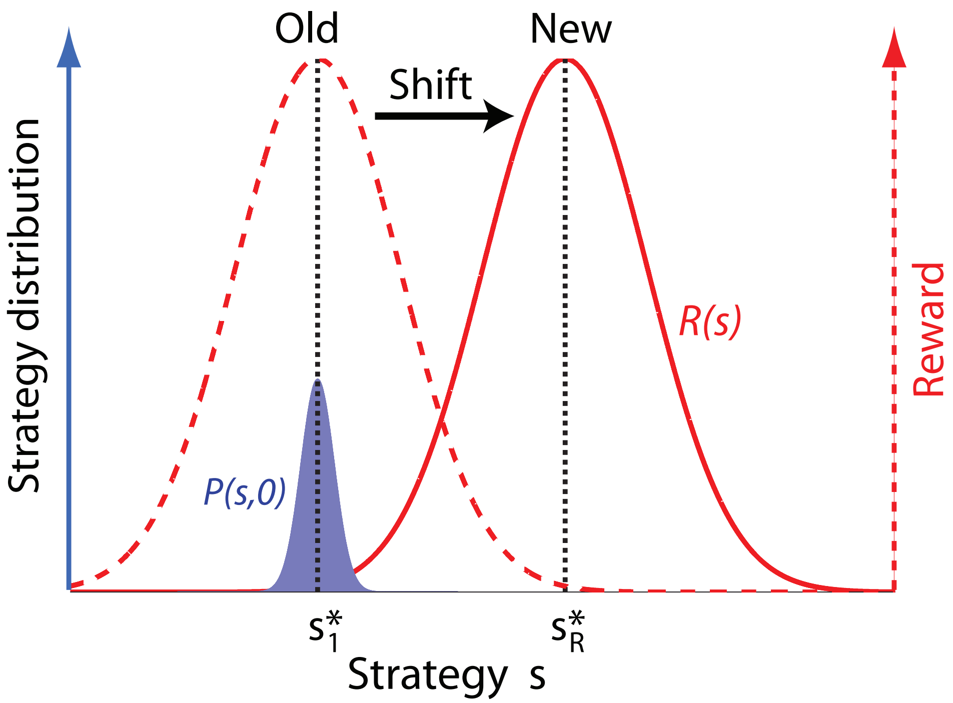

S Fig. 1: Schematic illustration of the shift-and-response scenario: The dashed and the solid red curves represent the reward kernel under the old (with a new best strategy s_1^*) the new (with a new best strategy s_R^* regimes, respectively. The blue curve represents the strategy distribution at the time that the shift occurs where it is centered about the old best strategy s_1^*.

reward kernel declines significantly for the strategies away from the best one, the population would always respond cohesively to the shift and never splits.

- The variance of the initial strategy distribution plays an important role during the time of shift; it determines how fast the responses is. The larger the variance, the faster the response and vice versa.

2 Graphical method and videos

As mentioned earlier, graphical method proposed by Muneepeerakul et al. uses Eq.2 and assumes that critical strategies $s_{m,t}$ which satisfy $\partial p(s,t)/\partial s|_{s_{m,t}} = 0$, locate either local maxima or local minima of the strategy distribution at time t. Performing the calculations, an identity equation for $s_{m,t}$ can be obtained as:

$$R'(s_{m,t}) = -\frac{p_0'(s_{m,t})}{tp_0(s_{m,t})}, \tag{3}$$

where the prime " $'$ " denotes the first derivative of the function with respect to s at $s_{m,t}$.

The top panel of each posted video shows how the bimodal strategy distribution $p(s,t)$ (blue) responds to a sudden shift under the new regime characterized by reward kernel $R(s,t) = R(s) = C\exp[-(s-s_R^*)^2/2\sigma^2]$ (red curve) with a new best strategy s_R^* and the number of emerging and disappearing peaks of the distribution. The bottom panel illustrates how the graphical method is used to locate the positions and number of emerging peaks during the response process. The red curve represents $R'(s)$ (the lhs of Eq.3), while the blue curve represents $-p_0'(s)/tp_0(s)$ (the rhs of Eq.3) where:

$$p_0(s) = \frac{w_1}{\sqrt{2\pi D_1^2}} \exp\left[-\frac{(s-s_1^*)^2}{2D_1^2}\right] + \frac{w_2}{\sqrt{2\pi D_2^2}} \exp\left[-\frac{(s-s_2^*)^2}{2D_2^2}\right]. \tag{4}$$

The intersections of the two curves, which could change over time, give the values of $s_{m,t}$ that represent either minima (trough) or maxima (emerging peak).

3 Results of different possible scenarios

Since the primary focus of this paper is on the transient dynamics, the reader is strongly encouraged to consider the video clips available online in conjunction with the analysis here (in which some snapshots from the video clips are shown[1]).

3.1 Middle-ground shift and symmetric variation

we assume that variations around the two peaks are equal, i.e., $D_1 = D_2$. Recall from Section 2.2 of the main text that D^2 controls the pace of response, and thus the two initial peaks are equally responsive in this case. It is therefore the distances from the new strategy, namely Δs_1^* and Δs_2^*, that determine the response dynamics. When both old most popular/ dominant strategies are of equal distances from the

[1]All video clips can be found in the online Supplementary Material - S1 File

new best one and above the critical threshold, i.e., $\Delta s_1^* = \Delta s_2^* > \Delta s_{crit}^*$, we observe that two new peaks appear simultaneously; as a result, four peaks coexist for a short period of time before the old peaks disappear and the new emerging peaks approach the new best strategy to form one single peak in the limit (see Fig.2-A and the related Video A). In this case, the initial peaks seem to behave *as if* they were two independent populations.

Breaking the symmetry in Δs^* but keeping them above the threshold, say, $\Delta s_1^* > \Delta s_2^* > \Delta s_{crit}^*$—$s_2^*$ is closer to s_R^* and thus of higher payoff than s_1^*—we still observe the emergence of two new peaks, but not simultaneously. As a result, four or three peaks may be observed during the transition period. In the peak emerging between s_R^* and s_2^* is formed first and quickly becomes the dominant peak. The second new peak, between s_R^* and s_1^*, may emerge later. Both new peaks continue to approach towards the new best strategy before they unite at the neighborhood of s_R^* (see Fig.2-B.1 and B.2 and the related Video B). If Δs_1^* is too large, the second new peak does not emerge at all, and only three peaks, at most, coexist (see Video C).

What happens if $\Delta s_1^* > \Delta s_{crit}^* > \Delta s_2^*$, i.e., only one peak is located beyond the critical threshold? The s_2^* peak would move cohesively towards s_R^*, while a new peak between s_R^* and s_1^* may or may not emerge, depending on how large Δs_1^* is. If Δs_1^* is too large, the s_1^* peak would simply disappear (see Video D); if s_1^* is not too far from s_R^*, a new peak will emerge, but it may not grow significantly as it approaches s_R^* due to the dominance of the peak cohesively moving from s_2^* (see Video E). If both peaks are located within the critical threshold, i.e., $\Delta s_1^*, \Delta s_2^* < \Delta s_{crit}^*$, both will move cohesively towards s_R^*. If $\Delta s_1^* = \Delta s_2^*$, it will take them a very long time for the two peaks to merge completely (see Video F). However, if one is closer than the other, it will reach s_R^*'s neighborhood earlier and dominate at the end (see Video G).

3.2 Middle-ground shift and asymmetric variation

What are the possible dynamical patterns when the variations around the two initial peaks differ, i.e., $D_1 \neq D_2$? Analogous to the previous section, we will now consider the response dynamics under different combinations of Δs_1^* and Δs_2^*. For ease of discussion, in the followings, let us suppose that $D_1 > D_2$; we can accordingly expect that the s_1^* peak would respond faster than its s_2^* counterpart.

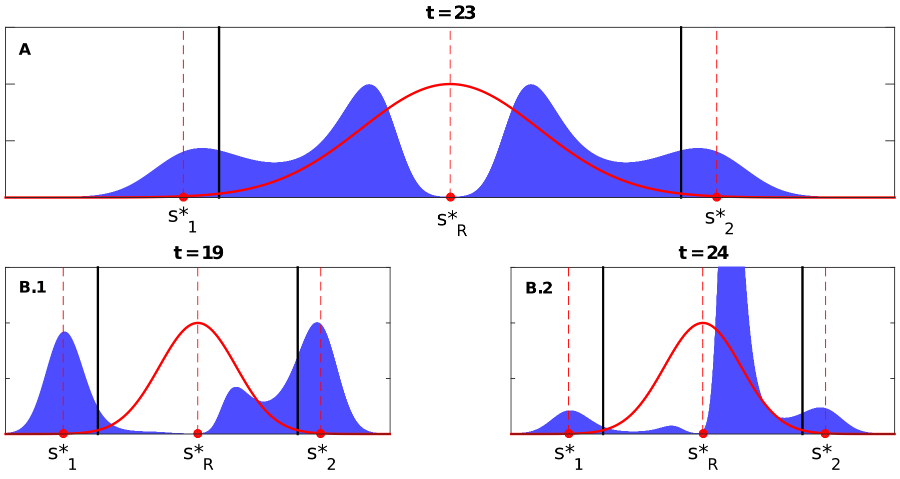

S Fig. 2: A) The panel illustrates the coexistence of four peaks at some time ($t = 23$) during the population response to a sudden middle-ground shift when $\Delta s_1^* = \Delta s_2^* = 0.3 > \Delta s_{crit}^* = 0.2598$ (where $s_1^* = 0.2$, $s_2^* = 0.8$, $s_R^* = 0.5$, and $\sigma = 0.1$ with $D_1 = D_2 = 0.04$), see Video A in supplementary S1 File. B) The bottom panels illustrates the different number of coexisting peaks at different times ($t = 19$ and 24) for the case when $\Delta s_1^* = 0.35 > \Delta s_2^* = 0.32 > \Delta s_{crit}^* = 0.2598$ (where $s_1^* = 0.15$, $s_2^* = 0.82$, $s_R^* = 0.5$, and $\sigma = 0.1$ with $D_1 = D_2 = 0.045$), (see Video B). Note that both cases are when the variations of the two peaks of the initial population distribution are equal to each other, i.e., $D_1 = D_2$. The dashed lines show the locations of s_1^*, s_2^*, and s_R^*, while the solid lines represent the the theoretically calculated threshold(s) for the single peak population distribution case, i.e., $\Delta s_{crit}^* = 3\sqrt{3}\sigma/2$.

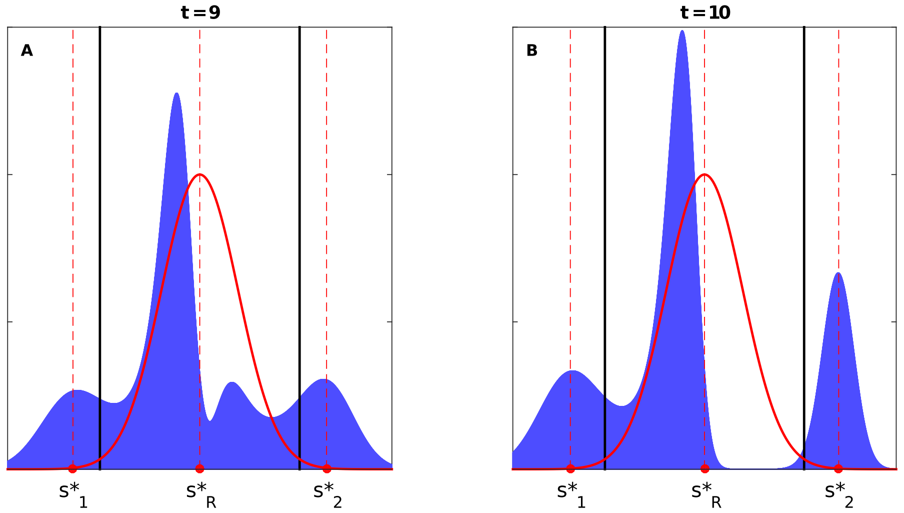

S Fig. 3: Snapshots show the coexistence of different peaks in the case of asymmetric variations of the peaks in the initial population distribution ($D_1 > D_2$) and when $\Delta s_1^* = \Delta s_2^* > \Delta s_{crit}^*$. A) Four peaks may coexist when $D_1 > D_2$ ($\Delta s_1^* = \Delta s_2^* = 0.33$ and $\Delta s_{crit}^* = 0.2589$, where $s_1^* = 0.17$, $s_2^* = 0.83$, $s_R^* = 0.5$, and $\sigma = 0.1$, with $D_1 = 0.08$ and $D_2 = 0.07$, see Video H). B) When the asymmetry in variations is stronger, i.e., $D_1 >> D_2$, only three peaks coexist ($\Delta s_1^* = \Delta s_2^* = 0.35$ and $\Delta s_{crit}^* = 0.2589$, where $s_1^* = 0.15$, $s_2^* = 0.85$, $s_R^* = 0.5$, and $\sigma = 0.1$, with $D_1 = 0.08$ and $D_2 = 0.04$, see Video I). The dashed lines show the locations of s_1^*, s_2^*, and s_R^*, while the solid lines represent the the theoretically calculated threshold(s) for the single peak population distribution case, i.e., $\Delta s_{crit}^* = 3\sqrt{3}\sigma/2$.

When $\Delta s_1^* = \Delta s_2^* > \Delta s_{crit}^*$, four peaks may be observed during the transition period. The new emerging peaks appear at different times; the first would emerge between s_R^* and s_1^* and dominate over the peak that appears later between s_R^* and s_2^* (see Fig.3-A and the related Video H). However, if $D_1 \gg D_2$, the first peak would grow quickly and move towards s_R^*. At this point, this new peak is of both better payoff and greater frequency; this suppresses the emergence of a second new peak as people would more likely adopt the strategies close to this new peak. As a result, only three peaks, at most, would coexist during the transition period (see Fig.3-B and the related Video I).

Consider now the case in which $\Delta s_1^* > \Delta s_{crit}^* > \Delta s_2^*$ (recall that $D_1 > D_2$). In this case, the s_2^* peak starts to move cohesively and slowly (due to a relatively low D) towards s_R^*. This moving peak *temporarily* dominates the strategy space while a new peak is emerging between s_R^* and s_1^*. However, the new peak may emerge very close to s_R^*—corresponding to better payoff—in which case it would eventually take over the moving s_2^* peak. The maximum of three peaks is observed in this case (see Fig.4-A.1 and

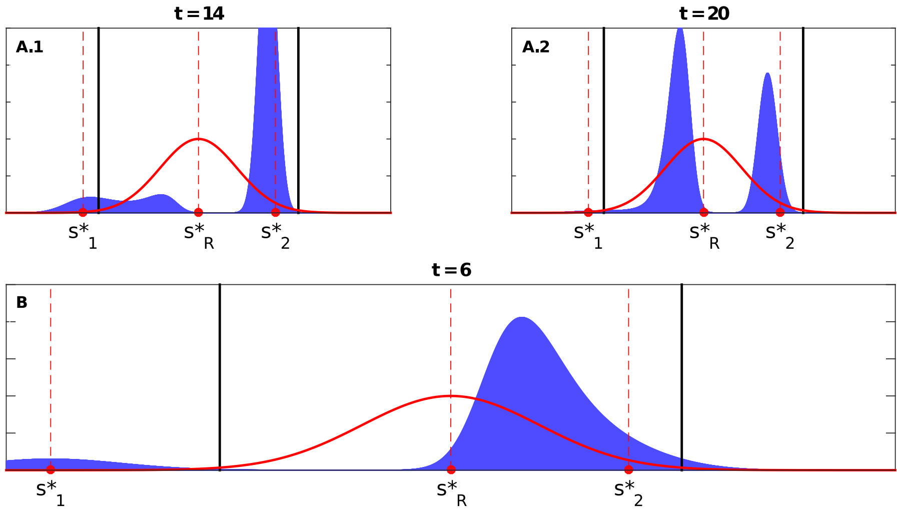

S Fig. 4: Snapshots show different number of coexisting peaks when $\Delta s_1^* > \Delta s_{crit}^* > \Delta s_2^*$ and $D_1 > D_2$. Top panels (A.1) show the coexistence of three peaks at $t = 14$ and (A.2) shows how the newly emerging peak near s_R^* dominates over the moving peak originated near s_2^* at $t = 20$, (where $\Delta s_1^* = 0.3$, $\Delta s_2^* = 0.2$, $\Delta s_{crit}^* = 0.2589$, with $s_1^* = 0.2$, $s_2^* = 0.7$, $s_R^* = 0.5$, and $\sigma = 0.1$, with $D_1 = 0.05$ and $D_2 = 0.02$, see Video J). B) When Δs_1^* is too large, the the moving peak originated near s_2^* will dominate and no emergence of a new peak near s_R^* (where $\Delta s_1^* = 0.45$, $\Delta s_2^* = 0.2$, $\Delta s_{crit}^* = 0.2589$, with $s_1^* = 0.05$, $s_2^* = 0.7$, $s_R^* = 0.5$, and $\sigma = 0.1$, with $D_1 = 0.08$ and $D_2 = 0.06$. see Video K). The dashed lines show the locations of s_1^*, s_2^*, and s_R^*, while the solid lines represent the the theoretically calculated threshold(s) for the single peak population distribution case, i.e., $\Delta s_{crit}^* = 3\sqrt{3}\sigma/2$.

A.2 and the related Video J). Now, If Δs_1^* is too large, there would be sufficient time for the moving peak from s_2^* to reach the high-payoff neighborhood of s_R^* such that the emergence of a new peak is suppressed (see Fig.4-B and the related Video K). In other words, there are no emerging peaks at all in this case.

When $\Delta s_1^*, \Delta s_2^* < \Delta s_{crit}^*$, both peak would move cohesively towards the new best strategy: which would dominate depends on the interplay between the values of D's and Δs^*'s. Video clips L, M, and N show a range of possible transient dynamics under this scenario.

3.3 Extreme shift and symmetric variation

In this section, we again consider the case where the variations around both peaks are equal but with an extreme shift, i.e., $s_R^* \notin [s_1^*, s_2^*]$; in what follows, we will assume that $s_1^* < s_2^* < s_R^*$ (see Fig. 2B in the main text). When both peaks are above the threshold ($\Delta s_1^*, \Delta s_2^* > \Delta s_{crit}^*$), a new peak emerges between

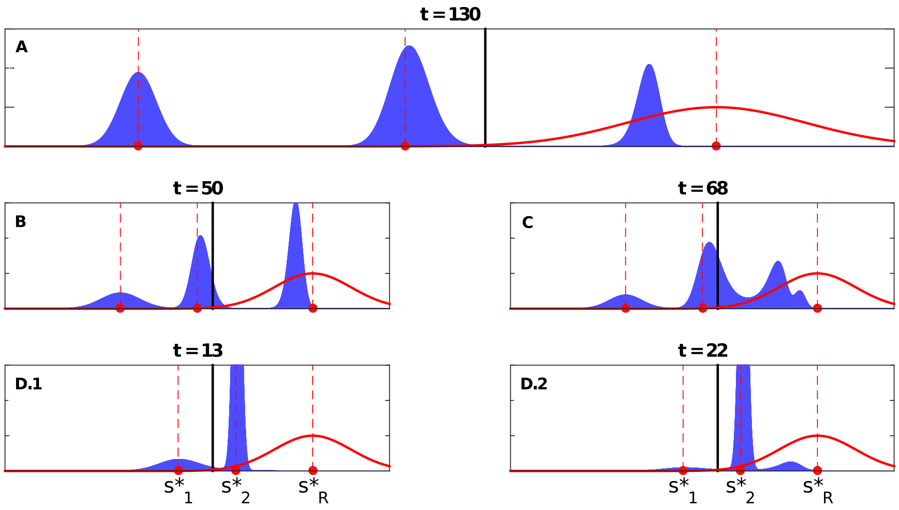

S Fig. 5: Snapshots of the coexisting peaks for the extreme case. A) The top panel shows the coexistence of three peaks when $\Delta s_1^* = 0.65, \Delta s_2^* = 0.35 > \Delta s_{crit}^* = 0.2598$, and with *symmetric* variations, i.e., $D_1 = D_2 = 0.2$ (where $s_1^* = 0.15$, $s_2^* = 0.45$, $s_R^* = 0.8$, $\sigma = 0.1$), see Video O. B) and C) Three or four peaks may coexist when $\Delta s_1^* = 0.5, \Delta s_2^* = 0.3 > \Delta s_{crit}^* = 0.2598$ but with *asymmetric* variations, i.e., $D_1 > D_2$ (where $s_1^* = 0.3$, $s_2^* = 0.5$, $s_R^* = 0.8$, $\sigma = 0.1$, with $D_1 = 0.05$ and $D_2 = 0.02$ for (B), and $D_1 = 0.0420$ and $D_2 = 0.0225$ for (C)), see R and S Videos, respectively. The bottom panels (D.1 and D.2) show the snapshots at different times when $\Delta s_1^* = 0.35 > \Delta s_{crit}^* = 0.2598 > \Delta s_2^* = 0.2$, and with *asymmetric* variations (where $s_1^* = 0.45$, $s_2^* = 0.6$, $s_R^* = 0.8$, $\sigma = 0.1$, with $D_1 = 0.05$ and $D_2 = 0.01$), see Video U. The dashed lines show the locations of s_1^*, s_2^*, and s_R^*, while the solid lines represent the the theoretically calculated threshold(s) for the single peak population distribution case, i.e., $\Delta s_{crit}^* = 3\sqrt{3}\sigma/2$.

s_2^* and s_R^*, grows quickly, and moves toward s_R^*, while the original peaks disintegrate. The fast growth of this new peak seems to prevent the emergence of any additional peaks. Hence, at most three peaks can coexist at the same time (see 5-A and the related Video O). When $\Delta s_2^* < \Delta s_{crit}^* < \Delta s_1^*$, the closer peak (i.e., the s_2^* peak) moves cohesively towards s_R^* and keeps growing and eventually dominates (see Video P). Similar dynamics can also be observed when $\Delta s_1^*, \Delta s_2^* < \Delta s_{crit}^*$ (Video Q).

3.4 Extreme shift and asymmetric variations

We now consider scenarios in which variations around the initial peaks not the same, i.e., $D_1 \neq D_2$. In this case, and given that the two peaks are on one side from the new best strategy, we consider two different cases: $D_1 > D_2$ and $D_1 < D_2$. As before, we will assume that $s_1^* < s_2^* < s_R^*$. And we will again

consider different cases of Δs_1^* and Δs_2^* with respect to Δs_{crit}^*.

Let us first consider the $D_1 > D_2$ cases. When $\Delta s_1^*, \Delta s_2^* > \Delta s_{crit}^*$, we may observe that a third peak appears between s_2^* and s_R^*, growing to dominance, while the original peaks are collapsing. Hence, three peaks may coexist at the same time (see Fig.5-B and the related Video R). It is possible, however, that for certain combinations of D's and Δs^*'s two new peaks may appear, and the one closer to s_R^* (and thus of higher payoff) eventually becomes the sole dominant peak. In this case, four peaks may coexist for some time during the transition period (see Fig.5-C and the related Video S).

When $\Delta s_2^* < \Delta s_{crit}^* < \Delta s_1^*$, different transient dynamics is observed. When Δs_1^* is relatively large, the s_1^* peak quickly erodes, while the s_2^* moves cohesively towards s_R^* and keeps growing in the process. In this case, no new peak is observed (Video T). But if Δs_1^* is just above Δs_{crit}^*, we may observe a new peak emerging between s_2^* and s_R^*, while the s_2^* peak moves slowly toward s_R^*. For a brief period of time, three peaks coexist, before the new emerging peak becomes dominant and the original peaks collapse (see 5-D.1 and D.2 and the related Video U).

Alternatively, it is also possible that the s_1^* peak collapses completely, leaving the s_2^* peak to be the only peak—*temporarily*—growing and drifting toward s_R^*. A new peak, however, suddenly appears even closer to s_R^* than the drifting one (see Fig.6 and the related Video V. This emerging peak grows rapidly and becomes the dominant one, and the moving s_2^* peak eventually collapses. Accordingly, in this case, we observe at most two peaks at a given time—with a brief period with only one dominant group of strategies.

When $\Delta s_1^*, \Delta s_2^* < \Delta s_{crit}^*$, one might expect that the two peaks move cohesively before the s_2^* peak dominates as it is closer to s_R^* and of higher payoff. This is, however, only true when the difference between D_1 and D_2 is relatively small (Video W). When the difference between the two D's is large, i.e., $D_1 \gg D_2$, we observe similar dynamics to that described in the previous paragraphs: the s_1^* peaks collapses, leaving the s_2^* peak as the sole dominant peak temporarily, but some time later, a new peak emerges close to s_R^* and eventually becomes the dominant one (see Video X).

What about the cases when $D_1 < D_2$? If $\Delta s_1^*, \Delta s_2^* > \Delta s_{crit}^*$, a third peak always appears closer to s_R^* and three peaks may coexist for some short period of time before the new peak becomes the dominant

one (Video Y). When $\Delta s_2^* < \Delta s_{crit}^* < \Delta s_1^*$, the s_2^* peak grows and moves cohesively toward s_R^*, while the s_1^* peak erodes and disappears completely; no new peak emerges. A similar scenario is observed when $\Delta s_1^*, \Delta s_2^* < \Delta s_{crit}^*$.

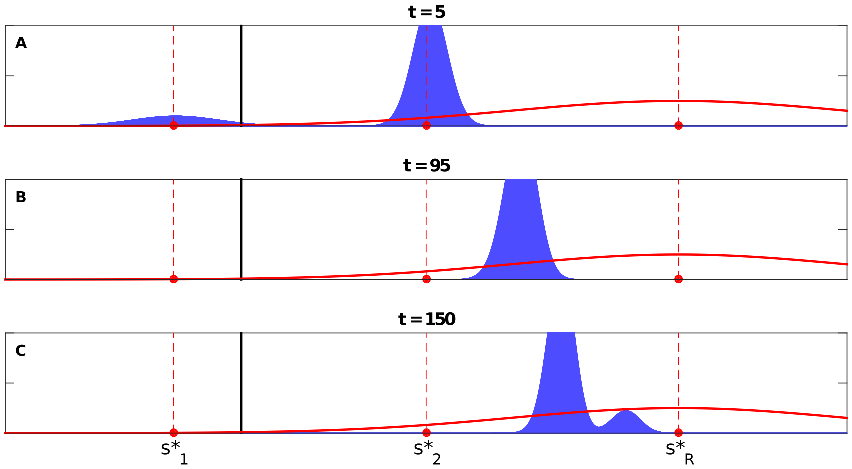

S Fig. 6: Different snapshots at different times for the case of $\Delta s_1^* > \Delta s_{crit}^* > \Delta s_2^*$ and with asymmetric variations (where $\Delta s_1^* = 0.6$, $\Delta s_2^* = 0.3$, $\Delta s_{crit}^* = 0.5196$, with $s_1^* = 0.2$, $s_2^* = 0.5$, $s_R^* = 0.8$, $\sigma = 0.2$, with $D_1 = 0.05$ and $D_2 = 0.02$, see Video V). Note that the peak initially around s_1^* disintegrates completely while the other peak dominates temporarily before a new peak emerges suddenly and dominates at the end. The dashed lines show the locations of s_1^*, s_2^*, and s_R^*, while the solid line represents the the theoretically calculated threshold for the single peak population distribution case, i.e., $\Delta s_{crit}^* = 3\sqrt{3}\sigma/2$.

References

[1] D. Fudenberg, D. K. Levine, The theory of learning in games, The MIT Press, 1998.

[2] G. P. Karev, On mathematical theory of selection: continuous time population dynamics, Journal of Mathematical Biology 60 (1) (2010) 107–129. doi:10.1007/s00285-009-0252-0.
URL http://dx.doi.org/10.1007/s00285-009-0252-0

[3] G. P. Karev, Replicator equations and the principle of minimal production of information, Bulletin of Mathematical Biology 72 (5) (2010) 1124–1142. doi:10.1007/s11538-009-9484-9.

URL http://dx.doi.org/10.1007/s11538-009-9484-9

[4] M. A. Nowak, Evolutionary Dynamics, Belknap/Harvard, 2006.

[5] P. Schuster, K. Sigmund, Replicator dynamics, Journal of Theoretical Biology 100 (3) (1983) 533–538. doi:10.1016/0022-5193(83)90445-9.
URL http://dx.doi.org/10.1016/0022-5193(83)90445-9

[6] P. D. Taylor, L. Jonker, Evolutionarily stable strategies and game dynamics, Math Biosci 40 (1978) 145–56.

[7] R. Muneepeerakul, M. R. Qubbaj, R. M. Aggarwal, J. M. Anderies, M. A. Janssen, Critical transition between cohesive and population-dividing responses to change, Journal of The Royal Society Interface 9 (77) (2012) 3303–3311. doi:10.1098/rsif.2012.0431.
URL http://dx.doi.org/10.1098/rsif.2012.0431

[8] C. P. Roca, J. A. Cuesta, A. Sánchez, Evolutionary game theory: Temporal and spatial effects beyond replicator dynamics, Physics of Life Reviews 6 (4) (2009) 208 – 249. doi:http://dx.doi.org/10.1016/j.plrev.2009.08.001.
URL http://www.sciencedirect.com/science/article/pii/S1571064509000256

Supplementary Material - Video Captions

Video A

Supplementary Video A: Middle ground shift and symmetric variation, i.e., $D_1 = D_2 = 0.04$, with $\Delta s_1^* = \Delta s_2^* = 0.30 > \Delta s_{Crit}^*$, where $s_1^* = 0.20$, $s_2^* = 0.80$, $s_R^* = 0.50$, and $\sigma = 0.10$. Theoretical value of $\Delta s_{Crit}^* = 3\sqrt{3}\sigma/2 = 0.2598$.

Video B

Supplementary Video B: Middle ground shift and symmetric variation, i.e., $D_1 = D_2 = 0.045$, with $\Delta s_1^* = 0.35 > \Delta s_2^* = 0.32 > \Delta s_{Crit}^*$, where $s_1^* = 0.15$, $s_2^* = 0.82$, $s_R^* = 0.50$, and $\sigma = 0.10$. Theoretical

value of $\Delta s^*_{Crit} = 3\sqrt{3}\sigma/2 = 0.2598$.

Video C

Supplementary Video C: Middle ground shift and symmetric variation, i.e., $D_1 = D_2 = 0.05$, with $\Delta s^*_1 = 0.42 >> \Delta s^*_2 = 0.3 > \Delta s^*_{Crit}$, where $s^*_1 = 0.08$, $s^*_2 = 0.80$, $s^*_R = 0.50$, and $\sigma = 0.08$. Theoretical value of $\Delta s^*_{Crit} = 3\sqrt{3}\sigma/2 = 0.2078$.

Video D

Supplementary Video D: Middle ground shift and symmetric variation, i.e., $D_1 = D_2 = 0.045$, with $\Delta s^*_1 = 0.42 > \Delta s^*_{Crit} > \Delta s^*_2 = 0.15$, where $s^*_1 = 0.05$, $s^*_2 = 0.65$, $s^*_R = 0.50$, and $\sigma = 0.10$. Theoretical value of $\Delta s^*_{Crit} = 3\sqrt{3}\sigma/2 = 0.2598$.

Video E

Supplementary Video E: Middle ground shift and symmetric variation, i.e., $D_1 = D_2 = 0.04$, with $\Delta s^*_1 = 0.28 > \Delta s^*_{Crit} > \Delta s^*_2 = 0.25$, where $s^*_1 = 0.22$, $s^*_2 = 0.75$, $s^*_R = 0.50$, and $\sigma = 0.10$. Theoretical value of $\Delta s^*_{Crit} = 3\sqrt{3}\sigma/2 = 0.2598$.

Video F

Supplementary Video F: Middle ground shift and symmetric variation, i.e., $D_1 = D_2 = 0.02$, with $\Delta s^*_{Crit} > \Delta s^*_1 = \Delta s^*_2 = 0.2$, where $s^*_1 = 0.30$, $s^*_2 = 0.70$, $s^*_R = 0.50$, and $\sigma = 0.10$. Theoretical value of $\Delta s^*_{Crit} = 3\sqrt{3}\sigma/2 = 0.2598$.

Video G

Supplementary Video G: Middle ground shift and symmetric variation, i.e., $D_1 = D_2 = 0.04$, with $\Delta s^*_{Crit} > \Delta s^*_1 = 0.22 > \Delta s^*_2 = 0.20$, where $s^*_1 = 0.28$, $s^*_2 = 0.70$, $s^*_R = 0.50$, and $\sigma = 0.10$. Theoretical value of $\Delta s^*_{Crit} = 3\sqrt{3}\sigma/2 = 0.2598$.

Video H

Supplementary Video H: Middle ground shift and asymmetric variation, i.e., $D_1 = 0.08 > D_2 = 0.07$, with $\Delta s_1^* = \Delta s_2^* = 0.33 > \Delta s_{Crit}^*$*, where $s_1^* = 0.17$, $s_2^* = 0.83$, $s_R^* = 0.50$, and $\sigma = 0.10$. Theoretical value of $\Delta s_{Crit}^* = 3\sqrt{3}\sigma/2 = 0.2598$.

Video I

Supplementary Video I: Middle ground shift and asymmetric variation, i.e., $D_1 = 0.08 >> D_2 = 0.04$, with $\Delta s_1^* = \Delta s_2^* = 0.35 > \Delta s_{Crit}^*$*, where $s_1^* = 0.15$, $s_2^* = 0.85$, $s_R^* = 0.50$, and $\sigma = 0.10$. Theoretical value of $\Delta s_{Crit}^* = 3\sqrt{3}\sigma/2 = 0.2598$.

Video J

Supplementary Video J: Middle ground shift and asymmetric variation, i.e., $D_1 = 0.05 > D_2 = 0.02$, with $\Delta s_1^* = 0.30 > \Delta s_{Crit}^* > \Delta s_2^* = 0.20$, where $s_1^* = 0.20$, $s_2^* = 0.70$, $s_R^* = 0.50$, and $\sigma = 0.10$. Theoretical value of $\Delta s_{Crit}^* = 3\sqrt{3}\sigma/2 = 0.2598$.

Video K

Supplementary Video K: Middle ground shift and asymmetric variation, i.e., $D_1 = 0.08 > D_2 = 0.06$, with $\Delta s_1^* = 0.45 > \Delta s_{Crit}^* > \Delta s_2^* = 0.20$, where $s_1^* = 0.05$, $s_2^* = 0.70$, $s_R^* = 0.50$, and $\sigma = 0.10$. Theoretical value of $\Delta s_{Crit}^* = 3\sqrt{3}\sigma/2 = 0.2598$.

Video L

Supplementary Video L: Middle ground shift and asymmetric variation, i.e., $D_1 = 0.04 > D_2 = 0.03$, with $\Delta s_{Crit}^* > \Delta s_1^* = 0.20 > \Delta s_2^* = 0.15$, where $s_1^* = 0.30$, $s_2^* = 0.65$, $s_R^* = 0.50$, and $\sigma = 0.10$. Theoretical value of $\Delta s_{Crit}^* = 3\sqrt{3}\sigma/2 = 0.2598$.

Video M

Supplementary Video M: Middle ground shift and asymmetric variation, i.e., $D_1 = 0.05 > D_2 = 0.03$, with $\Delta s_{Crit}^* > \Delta s_1^* = 0.20 > \Delta s_2^* = 0.10$, where $s_1^* = 0.30$, $s_2^* = 0.60$, $s_R^* = 0.50$, and $\sigma = 0.10$. Theoretical

value of $\Delta s^*_{Crit} = 3\sqrt{3}\sigma/2 = 0.2598$.

Video N

Supplementary Video N: Middle ground shift and asymmetric variation, i.e., $D_1 = 0.02 > D_2 = 0.01$, with $\Delta s^*_{Crit} > \Delta s^*_2 = 0.20 > \Delta s^*_1 = 0.15$, where $s^*_1 = 0.35$, $s^*_2 = 0.70$, $s^*_R = 0.50$, and $\sigma = 0.10$. Theoretical value of $\Delta s^*_{Crit} = 3\sqrt{3}\sigma/2 = 0.2598$.

Video O

Supplementary Video O: Extreme shift and symmetric variation, i.e., $D_1 = D_2 = 0.02$, with $\Delta s^*_1 = 0.65 > \Delta s^*_2 = 0.35 > \Delta s^*_{Crit}$, where $s^*_1 = 0.15$, $s^*_2 = 0.45$, $s^*_R = 0.80$, and $\sigma = 0.10$. Theoretical value of $\Delta s^*_{Crit} = 3\sqrt{3}\sigma/2 = 0.2598$.

Video P

Supplementary Video P: Extreme shift and symmetric variation, i.e., $D_1 = D_2 = 0.03$, with $\Delta s^*_1 = 0.35 > \Delta s^*_{Crit} > \Delta s^*_2 = 0.15$, where $s^*_1 = 0.25$, $s^*_2 = 0.45$, $s^*_R = 0.60$, and $\sigma = 0.10$. Theoretical value of $\Delta s^*_{Crit} = 3\sqrt{3}\sigma/2 = 0.2598$.

Video Q

Supplementary Video Q: Extreme shift and symmetric variation, i.e., $D_1 = D_2 = 0.02$, with $\Delta s^*_{Crit} > \Delta s^*_1 = 0.50 > \Delta s^*_2 = 0.25$, where $s^*_1 = 0.30$, $s^*_2 = 0.55$, $s^*_R = 0.80$, and $\sigma = 0.20$. Theoretical value of $\Delta s^*_{Crit} = 3\sqrt{3}\sigma/2 = 0.5196$.

Video R

Supplementary Video R: Extreme shift and asymmetric variation, i.e., $D_1 = 0.05 > D_2 = 0.02$, with $\Delta s^*_1 = 0.50 > \Delta s^*_2 = 0.30 > \Delta s^*_{Crit}$, where $s^*_1 = 0.30$, $s^*_2 = 0.50$, $s^*_R = 0.80$, and $\sigma = 0.10$. Theoretical value of $\Delta s^*_{Crit} = 3\sqrt{3}\sigma/2 = 0.2598$.

Video S

Supplementary Video S: Extreme shift and asymmetric variation, i.e., $D_1 = 0.042 > D_2 = 0.0225$, with $\Delta s_1^* = 0.50 > \Delta s_2^* = 0.30 > \Delta s_{Crit}^*$, where $s_1^* = 0.30$, $s_2^* = 0.50$, $s_R^* = 0.80$, and $\sigma = 0.10$. Theoretical value of $\Delta s_{Crit}^* = 3\sqrt{3}\sigma/2 = 0.2598$.

Video T

Supplementary Video T: Extreme shift and asymmetric variation, i.e., $D_1 = 0.050 > D_2 = 0.020$, with $\Delta s_1^* = 0.75 > \Delta s_2^* = 0.15 > \Delta s_{Crit}^*$, where $s_1^* = 0.05$, $s_2^* = 0.65$, $s_R^* = 0.80$, and $\sigma = 0.10$. Theoretical value of $\Delta s_{Crit}^* = 3\sqrt{3}\sigma/2 = 0.2598$.

Video U

Supplementary Video U: Extreme shift and asymmetric variation, i.e., $D_1 = 0.050 > D_2 = 0.020$, with $\Delta s_1^* = 0.35 > \Delta s_2^* = 0.20 > \Delta s_{Crit}^*$, where $s_1^* = 0.45$, $s_2^* = 0.60$, $s_R^* = 0.80$, and $\sigma = 0.10$. Theoretical value of $\Delta s_{Crit}^* = 3\sqrt{3}\sigma/2 = 0.2598$.

Video V

Supplementary Video V: Extreme shift and asymmetric variation, i.e., $D_1 = 0.050 > D_2 = 0.010$, with $\Delta s_1^* = 0.60 > \Delta s_2^* = 0.30 > \Delta s_{Crit}^*$, where $s_1^* = 0.20$, $s_2^* = 0.50$, $s_R^* = 0.80$, and $\sigma = 0.20$. Theoretical value of $\Delta s_{Crit}^* = 3\sqrt{3}\sigma/2 = 0.5196$.

Video W

Supplementary Video W: Extreme shift and asymmetric variation, i.e., $D_1 = 0.050 > D_2 = 0.030$, with $\Delta s_{Crit}^* > \Delta s_1^* = 0.50 > \Delta s_2^* = 0.20$, where $s_1^* = 0.30$, $s_2^* = 0.60$, $s_R^* = 0.80$, and $\sigma = 0.10$. Theoretical value of $\Delta s_{Crit}^* = 3\sqrt{3}\sigma/2 = 0.2598$.

Video X

Supplementary Video X: Extreme shift and asymmetric variation, i.e., $D_1 = 0.040 >> D_2 = 0.010$, with

$\Delta s^*_{Crit} > \Delta s^*_1 = 0.50 > \Delta s^*_2 = 0.20$, where $s^*_1 = 0.30$, $s^*_2 = 0.60$, $s^*_R = 0.80$, and $\sigma = 0.20$. Theoretical value of $\Delta s^*_{Crit} = 3\sqrt{3}\sigma/2 = 0.5196$.

Video Y

Supplementary Video Y: Extreme shift and asymmetric variation, i.e., $D_1 = 0.020 > D_2 = 0.040$, with $\Delta s^*_1 = 0.50 > \Delta s^*_2 = 0.30 > \Delta s^*_{Crit}$, where $s^*_1 = 0.30$, $s^*_2 = 0.50$, $s^*_R = 0.80$, and $\sigma = 0.10$. Theoretical value of $\Delta s^*_{Crit} = 3\sqrt{3}\sigma/2 = 0.2598$.
