# Supplementary material for: How Does a Divided Population Respond to Change?
Source: PLoS One. 2015 Jul 10;10(7):e0128121. doi: 10.1371/journal.pone.0128121 (PMC4498743; doi:10.1371/journal.pone.0128121)
Supplement: S1 File — (ZIP) [file pone.0128121.s001.zip › S1 File/Video Captions.pdf]

How Does a Divided Population Respond to Change?

Supplementary Material - Video Captions

Video A

Supplementary Video A: Middle ground shift and symmetric variation, i.e., $D_1 = D_2 = 0.04$, with $\Delta s_1^* = \Delta s_2^* = 0.30 > \Delta s_{Crit}^*$, where $s_1^* = 0.20$, $s_2^* = 0.80$, $s_R^* = 0.50$, and $\sigma = 0.10$. Theoretical value of $\Delta s_{Crit}^* = 3\sqrt{3}\sigma/2 = 0.2598$.

Video B

Supplementary Video B: Middle ground shift and symmetric variation, i.e., $D_1 = D_2 = 0.045$, with $\Delta s_1^* = 0.35 > \Delta s_2^* = 0.32 > \Delta s_{Crit}^*$, where $s_1^* = 0.15$, $s_2^* = 0.82$, $s_R^* = 0.50$, and $\sigma = 0.10$. Theoretical value of $\Delta s_{Crit}^* = 3\sqrt{3}\sigma/2 = 0.2598$.

Video C

Supplementary Video C: Middle ground shift and symmetric variation, i.e., $D_1 = D_2 = 0.05$, with $\Delta s_1^* = 0.42 >> \Delta s_2^* = 0.3 > \Delta s_{Crit}^*$, where $s_1^* = 0.08$, $s_2^* = 0.80$, $s_R^* = 0.50$, and $\sigma = 0.08$. Theoretical value of $\Delta s_{Crit}^* = 3\sqrt{3}\sigma/2 = 0.2078$.

Video D

Supplementary Video D: Middle ground shift and symmetric variation, i.e., $D_1 = D_2 = 0.045$, with $\Delta s_1^* = 0.42 > \Delta s_{Crit}^* > \Delta s_2^* = 0.15$, where $s_1^* = 0.05$, $s_2^* = 0.65$, $s_R^* = 0.50$, and $\sigma = 0.10$. Theoretical value of $\Delta s_{Crit}^* = 3\sqrt{3}\sigma/2 = 0.2598$.

Video E

Supplementary Video E: Middle ground shift and symmetric variation, i.e., $D_1 = D_2 = 0.04$, with $\Delta s_1^* = 0.28 > \Delta s_{Crit}^* > \Delta s_2^* = 0.25$, where $s_1^* = 0.22$, $s_2^* = 0.75$, $s_R^* = 0.50$, and $\sigma = 0.10$. Theoretical value of $\Delta s_{Crit}^* = 3\sqrt{3}\sigma/2 = 0.2598$.

Video F

Supplementary Video F: Middle ground shift and symmetric variation, i.e., $D_1 = D_2 = 0.02$, with $\Delta s_{Crit}^* > \Delta s_1^* = \Delta s_2^* = 0.2$, where $s_1^* = 0.30$, $s_2^* = 0.70$, $s_R^* = 0.50$, and $\sigma = 0.10$. Theoretical value of $\Delta s_{Crit}^* = 3\sqrt{3}\sigma/2 = 0.2598$.

Video G

Supplementary Video G: Middle ground shift and symmetric variation, i.e., $D_1 =$

$D_2 = 0.04$, with $\Delta s^*_{Crit} > \Delta s^*_1 = 0.22 > \Delta s^*_2 = 0.20$, where $s^*_1 = 0.28$, $s^*_2 = 0.70$, $s^*_R = 0.50$, and $\sigma = 0.10$. Theoretical value of $\Delta s^*_{Crit} = 3\sqrt{3}\sigma/2 = 0.2598$.

Video H

Supplementary Video H: Middle ground shift and asymmetric variation, i.e., $D_1 = 0.08 > D_2 = 0.07$, with $\Delta s^*_1 = \Delta s^*_2 = 0.33 > \Delta s^*_{Crit}$*, where $s^*_1 = 0.17$, $s^*_2 = 0.83$, $s^*_R = 0.50$, and $\sigma = 0.10$. Theoretical value of $\Delta s^*_{Crit} = 3\sqrt{3}\sigma/2 = 0.2598$.

Video I

Supplementary Video I: Middle ground shift and asymmetric variation, i.e., $D_1 = 0.08 >> D_2 = 0.04$, with $\Delta s^*_1 = \Delta s^*_2 = 0.35 > \Delta s^*_{Crit}$*, where $s^*_1 = 0.15$, $s^*_2 = 0.85$, $s^*_R = 0.50$, and $\sigma = 0.10$. Theoretical value of $\Delta s^*_{Crit} = 3\sqrt{3}\sigma/2 = 0.2598$.

Video J

Supplementary Video J: Middle ground shift and asymmetric variation, i.e., $D_1 = 0.05 > D_2 = 0.02$, with $\Delta s^*_1 = 0.30 > \Delta s^*_{Crit} > \Delta s^*_2 = 0.20$, where $s^*_1 = 0.20$, $s^*_2 = 0.70$, $s^*_R = 0.50$, and $\sigma = 0.10$. Theoretical value of $\Delta s^*_{Crit} = 3\sqrt{3}\sigma/2 = 0.2598$.

Video K

Supplementary Video K: Middle ground shift and asymmetric variation, i.e., $D_1 = 0.08 > D_2 = 0.06$, with $\Delta s^*_1 = 0.45 > \Delta s^*_{Crit} > \Delta s^*_2 = 0.20$, where $s^*_1 = 0.05$, $s^*_2 = 0.70$, $s^*_R = 0.50$, and $\sigma = 0.10$. Theoretical value of $\Delta s^*_{Crit} = 3\sqrt{3}\sigma/2 = 0.2598$.

Video L

Supplementary Video L: Middle ground shift and asymmetric variation, i.e., $D_1 = 0.04 > D_2 = 0.03$, with $\Delta s^*_{Crit} > \Delta s^*_1 = 0.20 > \Delta s^*_2 = 0.15$, where $s^*_1 = 0.30$, $s^*_2 = 0.65$, $s^*_R = 0.50$, and $\sigma = 0.10$. Theoretical value of $\Delta s^*_{Crit} = 3\sqrt{3}\sigma/2 = 0.2598$.

Video M

Supplementary Video M: Middle ground shift and asymmetric variation, i.e., $D_1 = 0.05 > D_2 = 0.03$, with $\Delta s^*_{Crit} > \Delta s^*_1 = 0.20 > \Delta s^*_2 = 0.10$, where $s^*_1 = 0.30$, $s^*_2 = 0.60$, $s^*_R = 0.50$, and $\sigma = 0.10$. Theoretical value of $\Delta s^*_{Crit} = 3\sqrt{3}\sigma/2 = 0.2598$.

Video N

Supplementary Video N: Middle ground shift and asymmetric variation, i.e., $D_1 = 0.02 > D_2 = 0.01$, with $\Delta s^*_{Crit} > \Delta s^*_2 = 0.20 > \Delta s^*_1 = 0.15$, where $s^*_1 = 0.35$, $s^*_2 = 0.70$, $s^*_R = 0.50$, and $\sigma = 0.10$. Theoretical value of $\Delta s^*_{Crit} = 3\sqrt{3}\sigma/2 = 0.2598$.

Video O

Supplementary Video O: Extreme shift and symmetric variation, i.e., $D_1 = D_2 = 0.02$, with $\Delta s^*_1 = 0.65 > \Delta s^*_2 = 0.35 > \Delta s^*_{Crit}$, where $s^*_1 = 0.15$, $s^*_2 = 0.45$, $s^*_R = 0.80$, and $\sigma = 0.10$. Theoretical value of $\Delta s^*_{Crit} = 3\sqrt{3}\sigma/2 = 0.2598$.

Video P

Supplementary Video P: Extreme shift and symmetric variation, i.e., $D_1 = D_2 =$

0.03, with $\Delta s_1^* = 0.35 > \Delta s_{Crit}^* > \Delta s_2^* = 0.15$, where $s_1^* = 0.25$, $s_2^* = 0.45$, $s_R^* = 0.60$, and $\sigma = 0.10$. Theoretical value of $\Delta s_{Crit}^* = 3\sqrt{3}\sigma/2 = 0.2598$.

Video Q

Supplementary Video Q: Extreme shift and symmetric variation, i.e., $D_1 = D_2 = 0.02$, with $\Delta s_{Crit}^* > \Delta s_1^* = 0.50 > \Delta s_2^* = 0.25$, where $s_1^* = 0.30$, $s_2^* = 0.55$, $s_R^* = 0.80$, and $\sigma = 0.20$. Theoretical value of $\Delta s_{Crit}^* = 3\sqrt{3}\sigma/2 = 0.5196$.

Video R

Supplementary Video R: Extreme shift and asymmetric variation, i.e., $D_1 = 0.05 > D_2 = 0.02$, with $\Delta s_1^* = 0.50 > \Delta s_2^* = 0.30 > \Delta s_{Crit}^*$, where $s_1^* = 0.30$, $s_2^* = 0.50$, $s_R^* = 0.80$, and $\sigma = 0.10$. Theoretical value of $\Delta s_{Crit}^* = 3\sqrt{3}\sigma/2 = 0.2598$.

Video S

Supplementary Video S: Extreme shift and asymmetric variation, i.e., $D_1 = 0.042 > D_2 = 0.0225$, with $\Delta s_1^* = 0.50 > \Delta s_2^* = 0.30 > \Delta s_{Crit}^*$, where $s_1^* = 0.30$, $s_2^* = 0.50$, $s_R^* = 0.80$, and $\sigma = 0.10$. Theoretical value of $\Delta s_{Crit}^* = 3\sqrt{3}\sigma/2 = 0.2598$.

Video T

Supplementary Video T: Extreme shift and asymmetric variation, i.e., $D_1 = 0.050 > D_2 = 0.020$, with $\Delta s_1^* = 0.75 > \Delta s_2^* = 0.15 > \Delta s_{Crit}^*$, where $s_1^* = 0.05$, $s_2^* = 0.65$, $s_R^* = 0.80$, and $\sigma = 0.10$. Theoretical value of $\Delta s_{Crit}^* = 3\sqrt{3}\sigma/2 = 0.2598$.

Video U

Supplementary Video U: Extreme shift and asymmetric variation, i.e., $D_1 = 0.050 > D_2 = 0.020$, with $\Delta s_1^* = 0.35 > \Delta s_2^* = 0.20 > \Delta s_{Crit}^*$, where $s_1^* = 0.45$, $s_2^* = 0.60$, $s_R^* = 0.80$, and $\sigma = 0.10$. Theoretical value of $\Delta s_{Crit}^* = 3\sqrt{3}\sigma/2 = 0.2598$.

Video V

Supplementary Video V: Extreme shift and asymmetric variation, i.e., $D_1 = 0.050 > D_2 = 0.010$, with $\Delta s_1^* = 0.60 > \Delta s_2^* = 0.30 > \Delta s_{Crit}^*$, where $s_1^* = 0.20$, $s_2^* = 0.50$, $s_R^* = 0.80$, and $\sigma = 0.20$. Theoretical value of $\Delta s_{Crit}^* = 3\sqrt{3}\sigma/2 = 0.5196$.

Video W

Supplementary Video W: Extreme shift and asymmetric variation, i.e., $D_1 = 0.050 > D_2 = 0.030$, with $\Delta s_{Crit}^* > \Delta s_1^* = 0.50 > \Delta s_2^* = 0.20$, where $s_1^* = 0.30$, $s_2^* = 0.60$, $s_R^* = 0.80$, and $\sigma = 0.10$. Theoretical value of $\Delta s_{Crit}^* = 3\sqrt{3}\sigma/2 = 0.2598$.

Video X

Supplementary Video X: Extreme shift and asymmetric variation, i.e., $D_1 = 0.040 >> D_2 = 0.010$, with $\Delta s_{Crit}^* > \Delta s_1^* = 0.50 > \Delta s_2^* = 0.20$, where $s_1^* = 0.30$, $s_2^* = 0.60$, $s_R^* = 0.80$, and $\sigma = 0.20$. Theoretical value of $\Delta s_{Crit}^* = 3\sqrt{3}\sigma/2 = 0.5196$.

Video Y

Supplementary Video Y: Extreme shift and asymmetric variation, i.e., $D_1 = 0.020 > D_2 = 0.040$, with $\Delta s_1^* = 0.50 > \Delta s_2^* = 0.30 > \Delta s_{Crit}^*$, where $s_1^* = 0.30$, $s_2^* = 0.50$, $s_R^* = 0.80$, and $\sigma = 0.10$. Theoretical value of $\Delta s_{Crit}^* = 3\sqrt{3}\sigma/2 = 0.2598$.
